# A CRISPR activation and interference toolkit for industrial *Saccharomyces cerevisiae* strain KE6-12

**DOI:** 10.1038/s41598-020-71648-w

**Published:** 2020-09-03

**Authors:** Elena Cámara, Ibai Lenitz, Yvonne Nygård

**Affiliations:** Division of Industrial Biotechnology, Department of Biology and Biological Engineering, Chalmers University of Technology, Kemivägen 10, 412 96 Gothenburg, Sweden

**Keywords:** Expression systems, Industrial microbiology, Metabolic engineering, Applied microbiology, Industrial microbiology, Biotechnology, Microbiology, Molecular biology

## Abstract

Recent advances in CRISPR/Cas9 based genome editing have considerably advanced genetic engineering of industrial yeast strains. In this study, we report the construction and characterization of a toolkit for CRISPR activation and interference (CRISPRa/i) for a polyploid industrial yeast strain. In the CRISPRa/i plasmids that are available in high and low copy variants, *dCas9* is expressed alone, or as a fusion with an activation or repression domain; *VP64*, *VPR* or *Mxi1*. The sgRNA is introduced to the CRISPRa/i plasmids from a double stranded oligonucleotide by in vivo homology-directed repair, allowing rapid transcriptional modulation of new target genes without cloning. The CRISPRa/i toolkit was characterized by alteration of expression of fluorescent protein-encoding genes under two different promoters allowing expression alterations up to ~ 2.5-fold. Furthermore, we demonstrated the usability of the CRISPRa/i toolkit by improving the tolerance towards wheat straw hydrolysate of our industrial production strain. We anticipate that our CRISPRa/i toolkit can be widely used to assess novel targets for strain improvement and thus accelerate the design-build-test cycle for developing various industrial production strains.

## Introduction

The yeast *Saccharomyces cerevisiae* is one of the most commonly used microorganisms for industrial applications ranging from wine and beer fermentations to the production of biofuels and high-value metabolites^1,2^. However, some of the current production processes are compromised by low yields and productivities, thus further optimization is required^3^. In particular, the production of second-generation bioethanol and other biochemicals from lignocellulosic biomass, which provides an alternative to oil-based chemicals, suffers from sub-optimal productivity^4^. During the hydrolysis of the raw material, inhibitory compounds (e.g. organic acids and aromatic aldehydes) are formed or released, compromising the microbial performance^5^. While quite some work has been done on elucidating genes required for tolerance, much less work has been done on improving tolerance towards stress by altering expression of genes^6^. Some previous studies demonstrated deletion^7–9^ or overexpression^10–14^ of endogenous genes to improve tolerance of *S. cerevisiae* towards inhibitors commonly found in lignocellulosic hydrolysates. However, most of the published work focuses on improving the tolerance of laboratory yeast strains that generally have weaker tolerance to stress^6^, while translation of beneficial modifications to more robust, industrial strains often is very challenging. The choice of yeast strain to be engineered is crucial for the successful implementation of the engineered phenotype in an industrial production process^15^.

Yeast strains used in industrial processes tend to be genetically diverse, since they usually arise from hybridization between different species^16^. Hence, they typically present aneuploidy, polyploidy or other chromosomal rearrangements^16–18^. This makes the strain development schemes for industrial yeast much more time-demanding and laborious compared to the engineering of well-studied laboratory strains^19^. In order to select for superior strain variants, industrial production strains are often subjected to directed evolution techniques such as random mutagenesis, protoplast fusion or genome shuffling^20^. Although these techniques are less dependent on biological information and available genetic tools, they suffer from being non-directional, having the risk of improving one trait to the detriment of others^20,21^.

In recent years, CRISPR/Cas technologies have become an indispensable tool for genome editing (reviewed by Pickar-Oliver and Gersbach^22^). These technologies are based on DNA-binding proteins (Cas endonucleases) and the single guide RNA (sgRNA) duplexes that can be easily programmed to precisely target and cleave a specific locus on double-stranded DNA. Recent studies have shown the applicability of this technology in industrial yeast strains, with the simultaneous disruption of two alleles of a gene or even several genes simultaneously without the introduction of a selection marker^23–26^. Still, the alteration of gene expression levels is often a more desirable metabolic engineering strategy compared to complete gene inactivation, or promoter swapping for overexpression^27^. Dynamic gene alteration allows optimizing trade-offs between growth and production^27^.

Alteration of gene expression by a nuclease-deficient form of Cas9, dCas9, often fused to an activation or repression domain, has been widely shown in both prokaryotes and eukaryotes^22^. This so-called CRISPR activation/interference (CRISPRa/i) technology is a way to modulate the expression of genes targeted by an sgRNA, allowing also the expression alteration of essential genes and the manipulation of multiple traits without altering the target sequence^28^. In laboratory yeast strains, the CRISPRa/i technology has been successfully employed to e.g. generate metabolic flux sensitivity maps^29^, identify chemical-genetic interactions^30^ and optimize different production pathways^31–33^. CRISPR based repression (CRISPRi) has been shown using dCas9 or dCas9 fused to repressor domains like KRAB (Krüppel associated box) or Mxi1 (reviewed by Jensen^34^). Similarly, gene activation by fusion of transcriptional activators, such as VP16, VP64 or VPR to dCas9 has been reported^34^.

In this study, we report the construction and characterization of a set of CRISPRa/i plasmids for the alteration of gene expression in a polyploid industrial yeast strain. In the CRISPRa/i plasmids, to which the sgRNA was introduced to its expression cassette by in vivo homology-directed repair, *dCas9* was expressed alone, or as a fusion with an activation or repression domain (i.e. *VP64*, *VPR* or *Mxi1*). The CRISPRa/i toolkit was used for the alteration of expression of fluorescent protein-encoding genes under two different promoters. Furthermore, we demonstrated the usability of the CRISPRa/i system by improving the tolerance towards growth in wheat straw hydrolysate, by targeting *SSK2*, a gene essential for tolerance to environmental stress^35^. We anticipate that our CRISPRa/i toolkit can be widely used to accelerate the design-build-test cycle for developing industrial production hosts.

## Results

### Modular design of the CRISPRa/i toolkit

**Figure 1:**
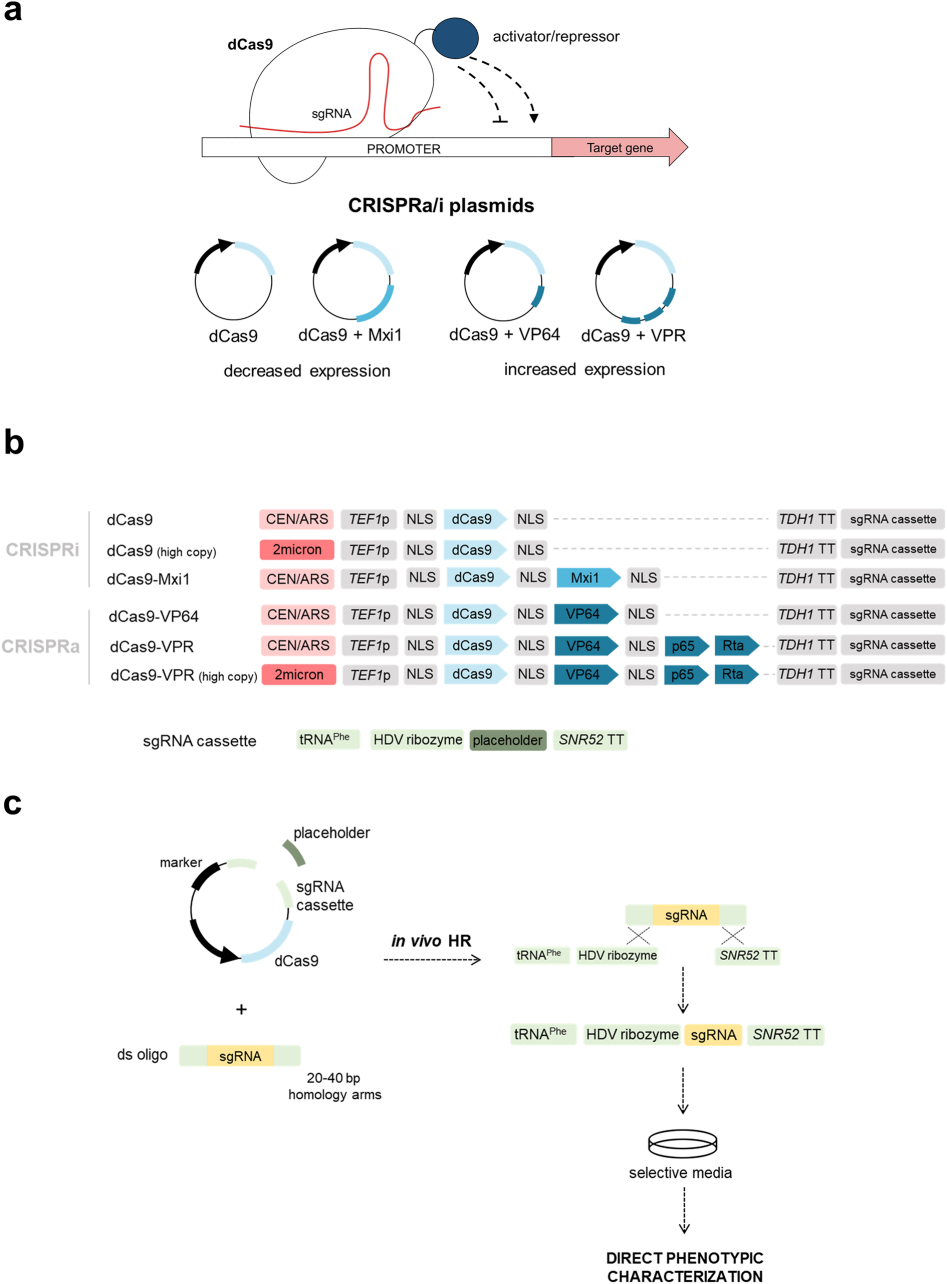
Design of the CRISPRa/i toolkit for transcriptional modulation. (**a**) The CRISPRa/i technology utilizes a catalytically inactive Cas9 (dCas9) to modulate the expression of genes targeted by an sgRNA. This can be further increased by fusing activation or repressor domains to dCas9. (**b**) Schematic representation of the CRISPRa/i components included in the CRISPRa/i plasmids and of the sgRNA expression cassette, present in all the plasmids. (**c**) The target-specific sgRNA from a double stranded oligonucleotide (ds oligo) was inserted into the CRISPRa/i plasmid through in vivo homologous recombination, allowing direct phenotypic characterization of strains with altered gene expression.

In order to obtain a CRISPRa/i toolkit for transcriptional modulation in industrial yeast, a set of plasmids was designed for the expression of *dCas9* and the sgRNA (Fig. 1a)*.* The dCas9 protein was expressed alone or fused to an activation or repression domain; the mammalian transcriptional repressor Mxi1^36^, the VP64 activation domain containing 4 tandem copies of Herpes Simplex Viral Protein 16^37^ or VPR, a fusion of three different activation domains; VP64, p65, and Rta^38^.

For all the CRISPRa/i plasmids, *dCas9* was cloned under the strong *TEF1* promoter and flanked by the SV40 nuclear localization sequence (Fig. 1b). The sgRNA cassette consisted of the phenylalanine tRNA promoter and an HDV ribozyme prior to a placeholder for the sgRNA, followed by the sgRNA scaffold and an *SNR52* terminator^39^ (Fig. 1b). To insert the 20 bp specific sgRNA sequence targeting a gene of interest, the CRISPRa/i plasmids were digested at the placeholder prior to the yeast transformation. Therefore, the placeholder fragment was replaced by the sgRNA by in vivo homologous recombination (Fig. 1c).

**Figure 2:**
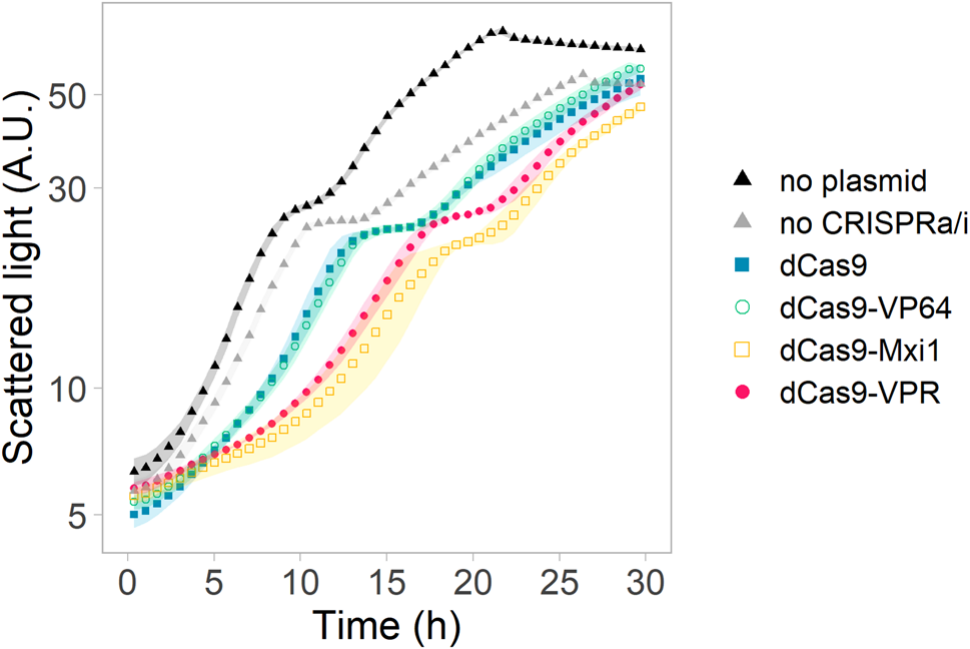
Growth of KE6-12-Ruby strains carrying different CRISPRa/i plasmids expressing *dCas9* (blue solid squares), *dCas9-VP64* (open turquoise circles)*, dCas9-Mxi1* (open yellow squares)*, dCas9-VPR* (solid pink circles) or an empty plasmid lacking the *dCas9* or sgRNA expression cassette (no CRISPRa/i strain, grey triangles). KE6-12-Ruby was added as a control to evaluate the plasmid effect (no plasmid strain, black triangles). The strains were grown in microbioreactors in YPD medium supplemented with geneticin for plasmid maintenance, and without antibiotic for the no plasmid strain. Data obtained from three biological replicates; shadowed regions show the standard deviation.

In order to assess the impact of the CRISPRa/i toolkit on growth of the strains, strains expressing *dCas9* or the *dCas9*-fusions (Fig. 1a) but no sgRNA were compared to a control strain carrying an empty plasmid lacking any *dCas9* expression cassette (Fig. 2 and Supplementary Table S1). In the strains expressing *dCas9-Mxi1* or *dCas9-VPR*, both the lag phase and the generation time were significantly (*p* < 0.05) increased compared to strains carrying an empty plasmid. Also the strain carrying an empty plasmid grew slightly slower than the parental strain (Fig. 2).

### CRISPRa/i demonstration by changing expression of a fluorescence protein

**Figure 3:**
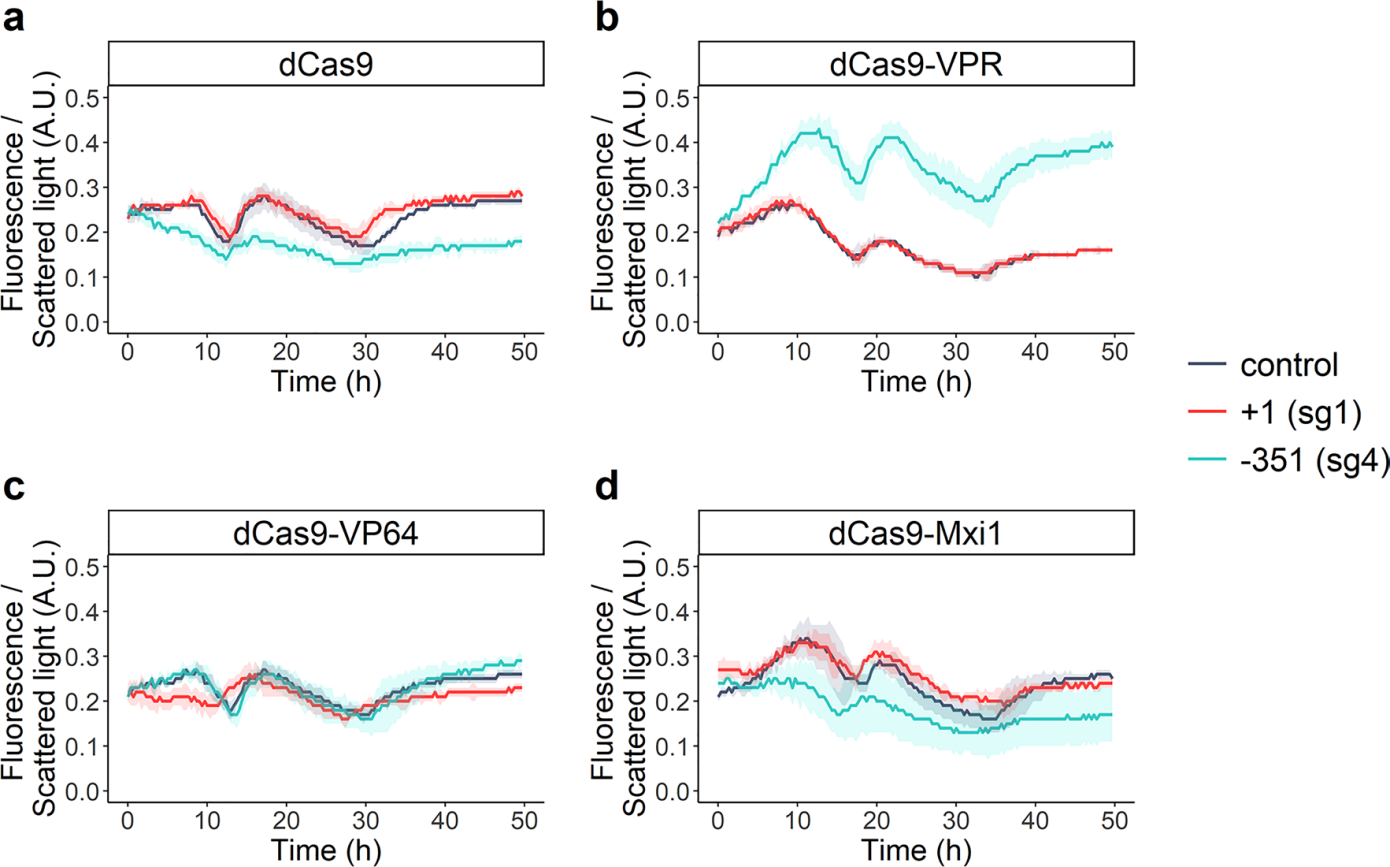
CRISPRa/i based change in expression of a fluorescent protein, measured over time in microbioreactors. Normalized fluorescence of strains expressing *dCas9* (**a**), *dCas9-VPR* (**b**), *dCas9-VP64* (**c**) or *dCas9-Mxi1* (**d**) and sgRNAs targeting a region at + 1 (sg1; red line) or − 351 (sg4; turquoise line) bp relative to the TSS or the CRISPRa/i plasmid with a placeholder (control; grey line). Data obtained from three biological replicates; shadowed regions show the standard deviation.

An expression cassette containing the red fluorescent protein-encoding gene *mRuby2* under *TDH3*p was put into the *PDR12* locus of the industrial strain KE6-12, a polyploid strain optimized for xylose consumption and ethanol production from lignocellulosic hydrolysates^40,41^, resulting in a strain named KE6-12-Ruby (Supplementary Fig. S1). Different CRISPRa/i plasmids and sgRNAs targeting the *TDH3*p promoter driving *mRuby2*, at positions + 1 or − 351 bp relative to the transcription starting site (TSS), were transformed into KE6-12-Ruby and the fluorescence of the strains was followed over time (Fig. 3 and Supplementary Fig. S2). Whereas no change in expression was observed when targeting position + 1, a significant (*p* = 6.5E−03) downregulation of 30% was obtained at the end of the culture when expressing *dCas9* together with the sgRNA targeting a region at − 351 bp (Fig. 3a). In strains expressing this sgRNA and *dCas9-VPR* a significant (*p* = 7.6E−03) increase of 144% in fluorescence was measured at the end of the culture (Fig. 3b). In contrast, in strains expressing *dCas9-VP64* or *dCas9-Mxi1* and the same sgRNA, no significant changes in expression were observed (Fig. 3c, d).

### Characterization of the CRISPRa/i toolkit

**Figure 4:**
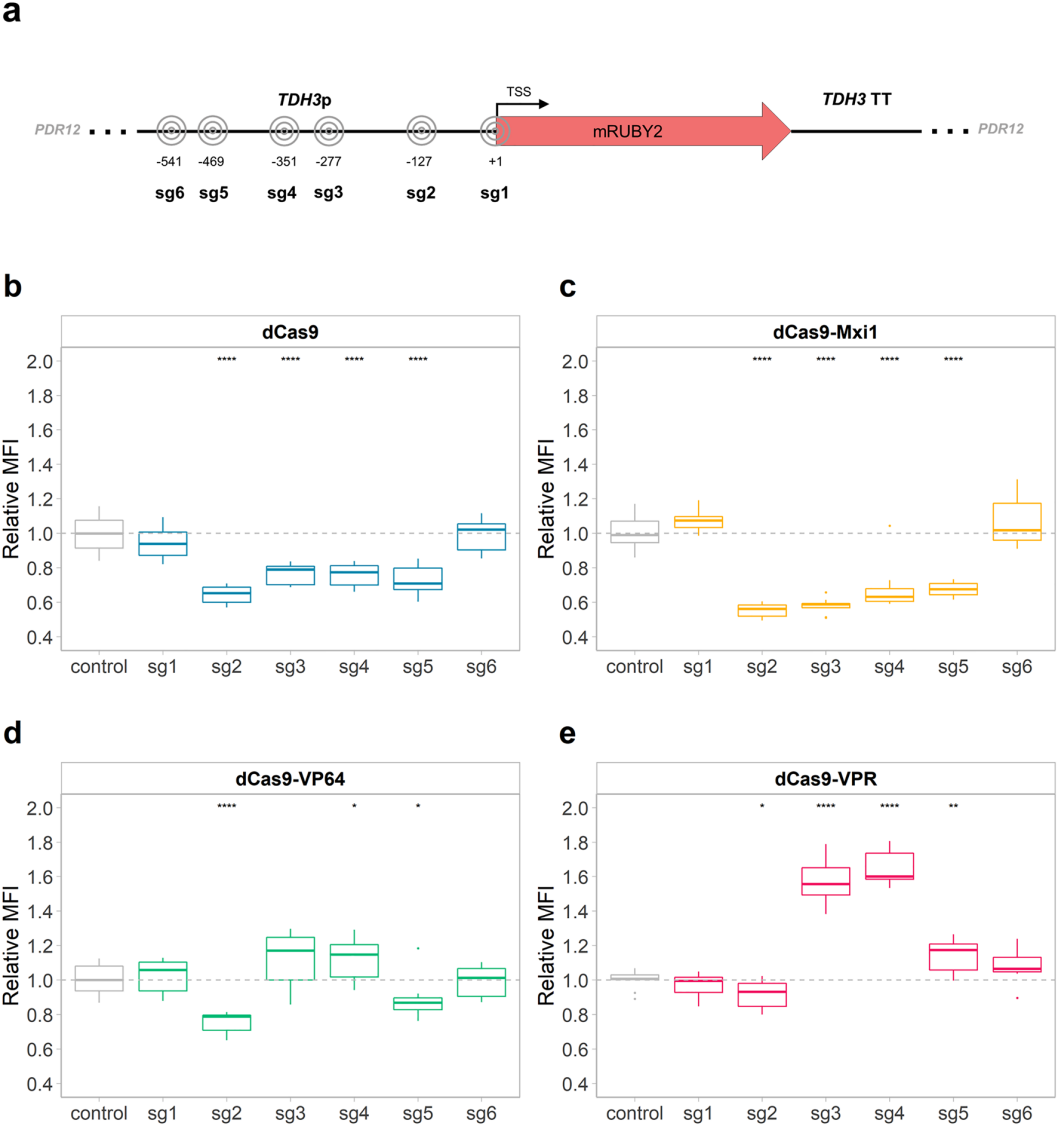
Modulation of *mRuby2* fluorescence by different CRISPRa/i plasmids. (**a**) Six sgRNAs targeting different loci of *TDH3*p were tested. (**b**)–(**e**) Relative MFI of strains expressing *dCas9* (**b**), *dCas9-Mxi1* (**c**), *dCas9-VP64* (**d**) and *dCas9-VPR* (**e**) together with either of the sgRNAs (sg1–6) or the placeholder (control). The box plots show the relative fluorescence, compared to KE6-12-Ruby expressing the corresponding CRISPRa/i plasmid with the placeholder (control, grey dashed line). Data obtained from three biological and three technical replicates. The line dividing the box represents the median of the data, the whiskers indicate the data outside the middle 50% and the outliers are shown as individual points. Statistical significance represented as “*” for *p* ≤ 0.05, “**” for *p* ≤ 0.01, “***” for *p* ≤ 0.001 and “****” for *p* ≤ 0.0001.

The impact of the sgRNA target region on the transcriptional modulation was evaluated using 6 different sgRNAs, targeting *TDH3p* at regions between − 541 and + 1 bp relative to the TSS (Fig. 4a). The mean fluorescence intensity (MFI) of the strains expressing *mRuby2* was measured by flow cytometry from samples taken after 24 h of cultivation (Fig. 4b–e), during exponential growth (Supplementary Fig. S2).

In strains expressing *dCas9* alone, the fluorescence of *mRuby2* was significantly reduced when targeting positions between − 127 and − 469 bp (sg2–5, *p* < 0.05) (Fig. 4b), reaching a maximal decrease of 35% with sg2. Likewise, expression of sg2, sg3, sg4, or sg5 together with *dCas9-Mxi1* led to a reduction in fluorescence (Fig. 4c). When targeting positions at − 127 or − 277 bp, the repression was increased by 10 or 18% in strains expressing *dCas9-Mxi1* compared to strains expressing *dCas9* alone (Fig. 4b, c). Strains expressing *dCas9-VP64* and sg4 showed a 10% increase in fluorescence compared to the no sgRNA control (Fig. 4d). In contrast, a decrease in *mRuby2* expression was obtained with sg2 and sg5. Strains expressing *dCas9-VPR* and sg3, sg4 or sg5 displayed an increase in fluorescence, with sg3 and sg4 targeting positions at − 277 or − 351 bp leading to highest activation, displaying an increase in fluorescence of 57% or 65%, respectively (Fig. 4e). The strain expressing *dCas9-VPR* and sg2 displayed a significant (*p* = 0.02) decrease in expression of *mRuby2* (Fig. 4e). Strains expressing any of the CRISPRa/i plasmids and sgRNAs sg1 or sg6 showed no change in expression compared to the control strain with a placeholder in the sgRNA cassette.

**Figure 5:**
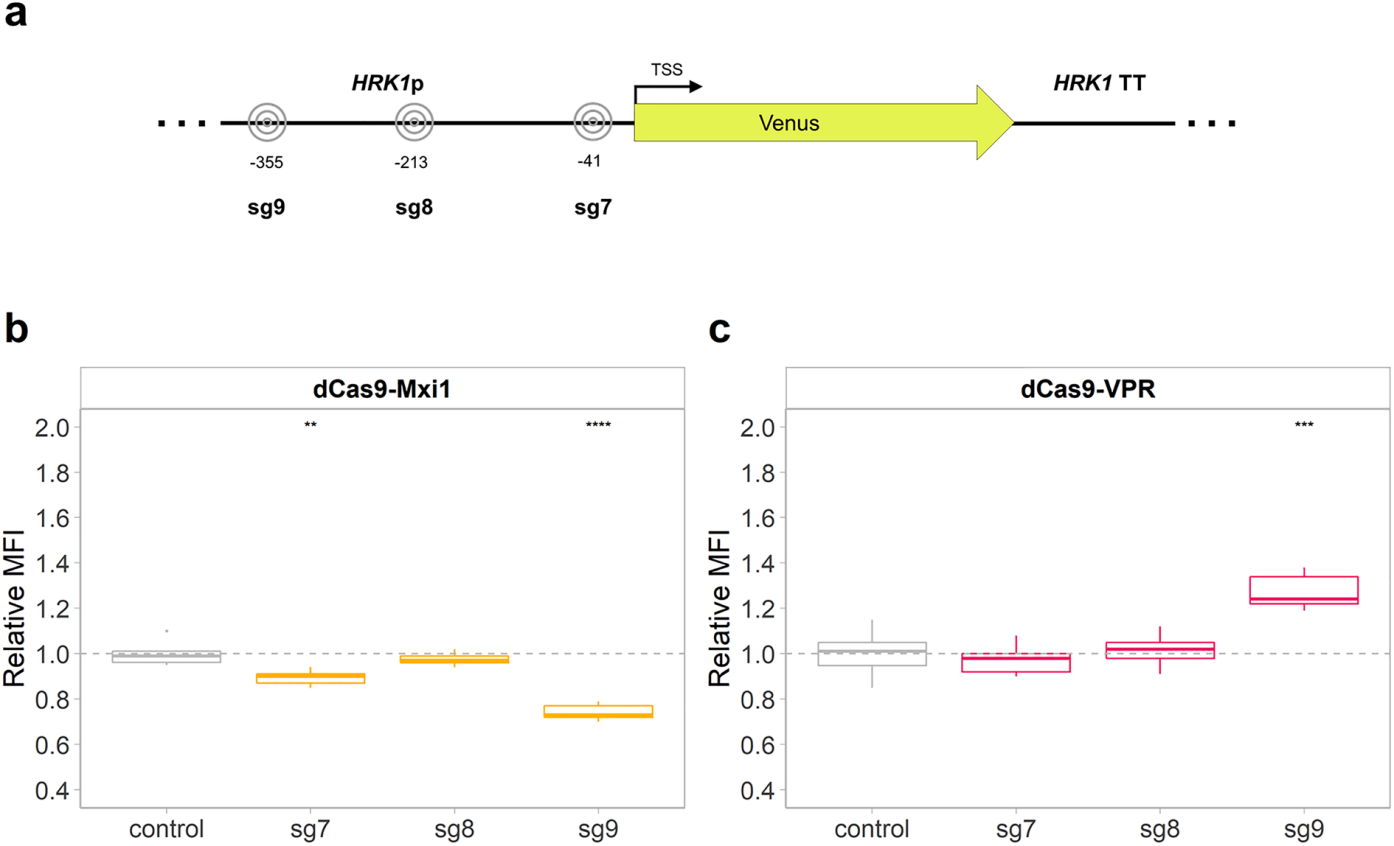
Modulation of fluorescence of *Venus* expressed under *HRK1*p by CRISPRa/i. (**a**) Three sgRNAs targeting different loci of *HRK1*p were tested. (**b**)–(**c**) Relative MFI of strains expressing *dCas9-Mxi1* (**b**), and *dCas9-VPR* (**c**) together with either of the sgRNAs (sg7–9) or the placeholder (control). The box plots show the relative fluorescence, compared to KE6-12-Ruby-Venus expressing the corresponding CRISPRa/i plasmid with the placeholder (control, grey dashed line). Data obtained from three biological and three technical replicates. The line dividing the box represents the median of the data, the whiskers indicate the data outside the middle 50% and the outliers are shown as individual points. Statistical significance represented as “*” for *p* ≤ 0.05, “**” for *p* ≤ 0.01, “***” for *p* ≤ 0.001 and “****” for *p* ≤ 0.0001.

To further test the applicability of the CRISPRa/i toolkit, the most effective plasmids were tested with sgRNAs targeting a presumably weaker promoter^42^, *HRK1*p. In KE6-12-Ruby-Venus all native copies of *HRK1* were replaced by a gene encoding the yellow fluorescent protein *Venus* (Fig. 5a and Supplementary Fig. S3). Three sgRNAs targeting positions at regions − 355, − 213 or − 40 bp relative to the TSS were tested, the most impactful being sg9, hybridizing at − 355 bp (Fig. 5b, c). The strain expressing *dCas9-Mxi1* and sg9 showed a repression of fluorescence by 26% (Fig. 5b) whereas the strain expressing *dCas9-VPR* showed a 27% increase in expression of *Venus*.

### High copy variants of the CRISPRa/i plasmids

**Figure 6:**
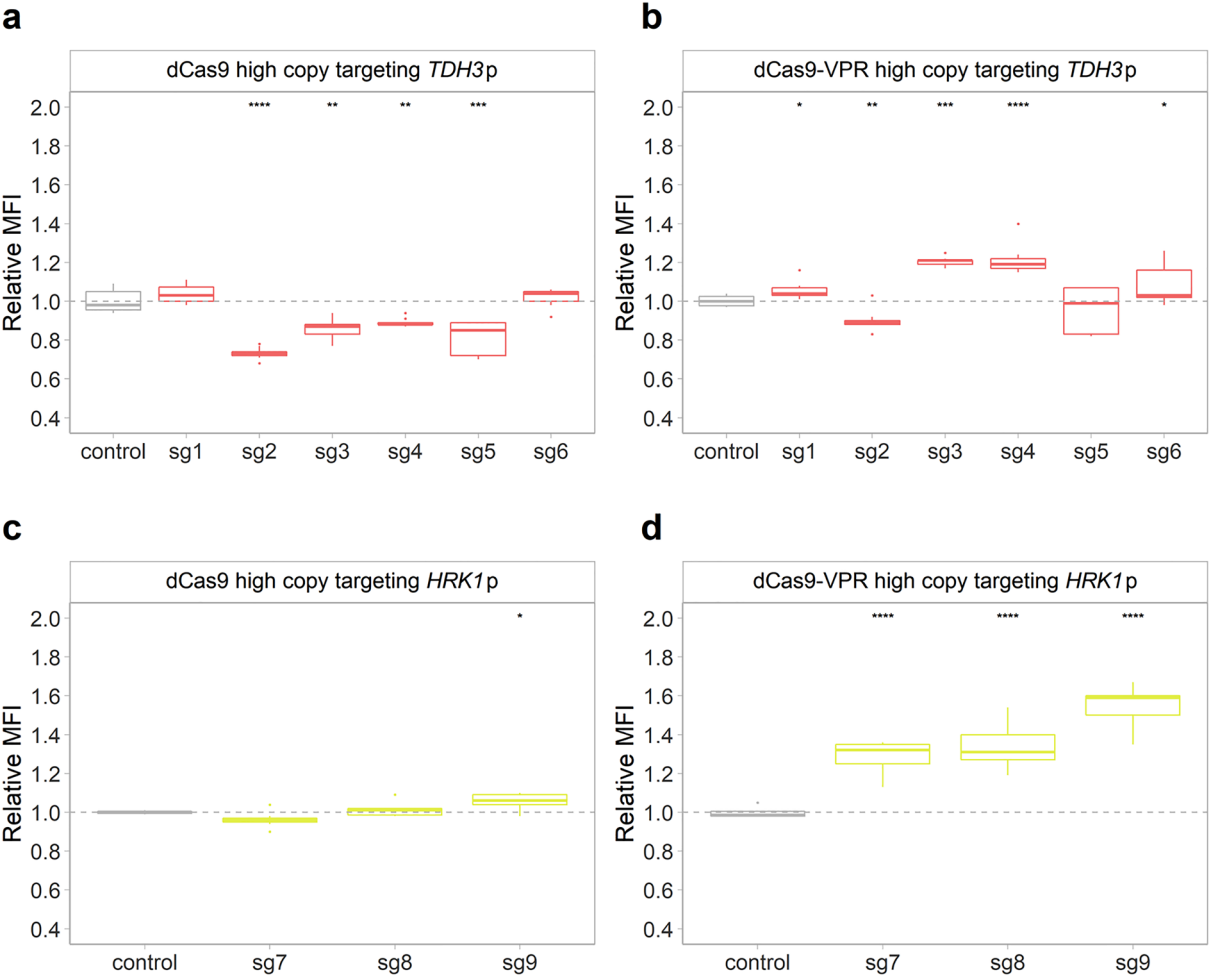
Modulation of fluorescence using high copy CRISPRa/i plasmids. (**a**) Relative MFI of strains expressing *dCas9* (**a**), or *dCas9-VPR* (**b**) together with either of the sgRNAs targeting *TDH3*p (sg1–6) or *HRK1*p (sg7–9) or the placeholder (control). The box plots show the relative fluorescence, compared to KE6-12-Ruby (**a**, **b**) or KE6-12-Ruby-Venus (**c**, **d**) expressing the corresponding high copy CRISPRa/i plasmid with the placeholder (control, grey dashed line). Data obtained from three biological and three technical replicates. The line dividing the box represents the median of the data, the whiskers indicate the data outside the middle 50% and the outliers are shown as individual points. Statistical significance represented as “*” for *p* ≤ 0.05, “**” for *p* ≤ 0.01, “***” for *p* ≤ 0.001 and “****” for *p* ≤ 0.0001.

In an attempt to increase the impact of the CRISPRa/i system, high copy variants of the CRISPRa/i plasmids expressing *dCas9* or *dCas9-VPR*, were used for targeting *TDH3*p in the KE6-12-Ruby strain or *HRK1*p in the KE6-12-Ruby-Venus strain. Nonetheless, the repression of m*Ruby2* expression was similar when using centromeric, low copy (Fig. 4b) or high copy plasmids (Fig. 6a) expressing *dCas9* and sg2, sg3, sg4 or sg5 (Supplementary Table S2). Similarly, expressing *dCas9* and sg7, sg8 or sg9 from the high copy plasmid led to strains with *Venus* expression similar to that of the corresponding centromeric plasmid carrying strains (Figs. 5a, 6c and Supplementary Table S3).

CRISPRa strains expressing *dCas9-VPR* and sg3 or sg4 from high copy plasmids (Fig. 6b) showed a 20% increase in expression of *mRuby2*, which was a threefold lower activation compared to expressing *dCas9-VPR* and the same sgRNAs from the low copy plasmid (Fig. 4e). Conversely, strains carrying the high copy CRISPRa plasmid expressing *dCas9-VPR* and sg7, sg8 or sg9 showed an increase in expression of *Venus* of 29, 34 and 56%, respectively (Fig. 6d). Nonetheless, strains with the high copy CRISPRa plasmid expressing *dCas9-VPR* displayed a serious growth defect (Supplementary Table S1).

### Application of the CRISPRa/i toolkit to improve tolerance towards lignocellulosic hydrolysate

Four genes reported to be involved in tolerance towards inhibitors found in lignocellulosic hydrolysate, *HRK1*^43^, *SSK2*^35^, *ISC1*^44^ and *BDH2*^45^, were selected as targets for applying the CRISPRa/I toolkit. Strains with 3 different sgRNAs were designed for locating dCas9 or dCas9-VPR to the promoter regions of these genes were tested in minimal medium with added inhibitors (Supplementary Fig. S4). Strains with sgRNAs targeting the promoters of *ISC1* or *BDH2* performed the same or worse than the strain with no sgRNA (Supplementary Fig. S4). Two sgRNAs targeting *HRK1* led to higher biomass yield whereas one of the sgRNAs tested resulted in a slower growth in medium supplemented with acetate (Supplementary Fig. S4). However, all strains expressing dCas9 and sgRNAs targeting *SSK2* grew better in medium supplemented with 20 mM furfural (Supplementary Fig. S4) and were therefore tested in lignocellulosic hydrolysate.

**Figure 7:**
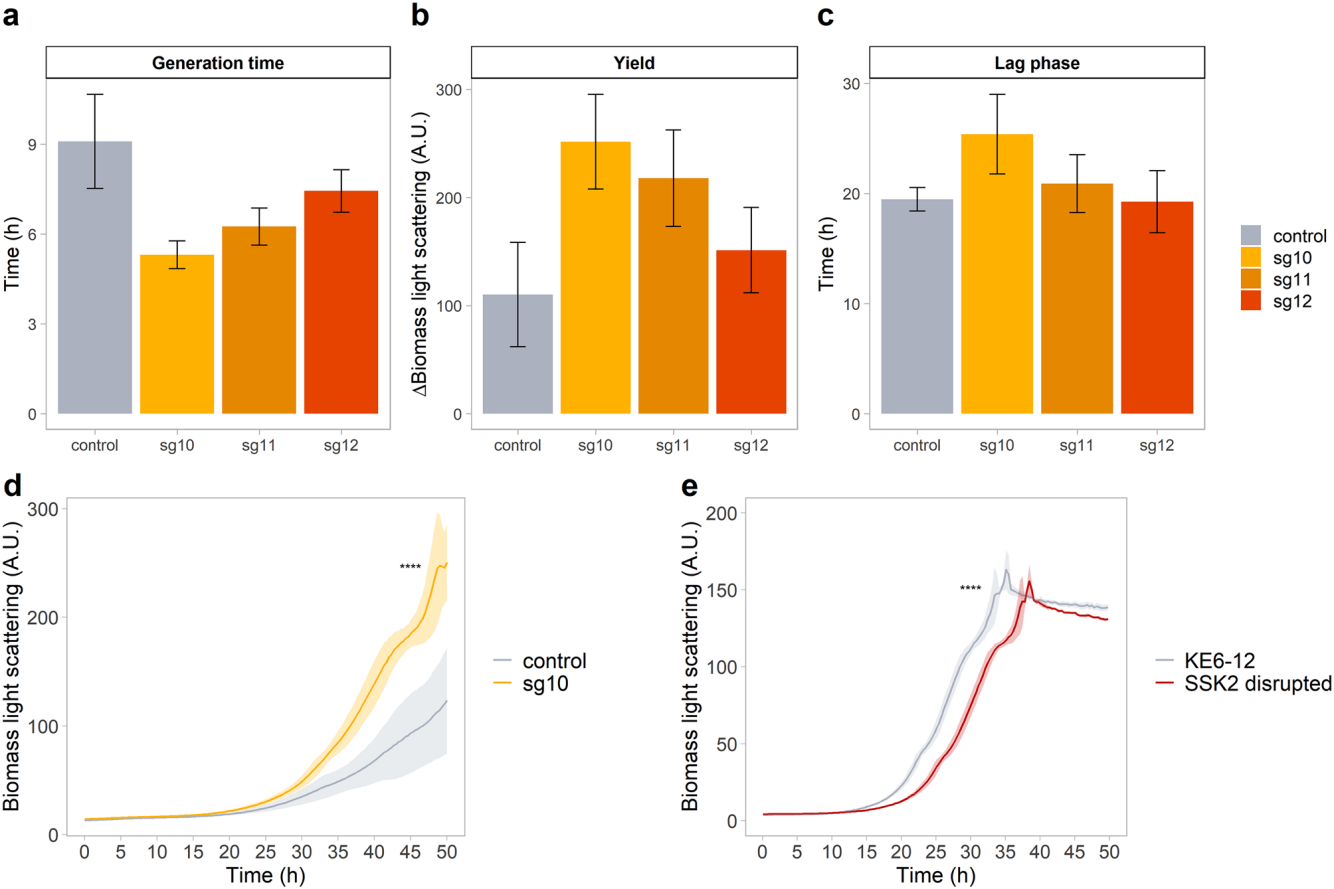
Application of CRISPRi for improving growth in wheat straw hydrolysate. (**a**) generation time, (**b**) biomass yield and (**c**) lag phase of KE6-12 expressing *dCas9* and different sgRNAs targeting *SSK2*p. (**d**) Growth of KE6-12 strain expressing *dCas9* and sg10 or the placeholder. (**e**) Growth of KE6-12 strain compared to KE6-12 with *SSK2* gene disrupted. The strains were grown in microbioreactors in 64% wheat straw hydrolysate for 50 h. Data obtained from three biological replicates (**a**–**d**) or three technical replicates (**e**) are expressed as mean ± standard deviation (SD). Statistical significance represented as “*” for *p* ≤ 0.05, “**” for *p* ≤ 0.01, “***” for *p* ≤ 0.001 and “****” for *p* ≤ 0.0001.

Downregulation of *SSK2* has previously been suggested to improve tolerance towards furfural^35^. Therefore, targeting *SSK2*p with the CRISPRi system, using either *dCas9* or *dCas9-Mxi1* expressing plasmids were tested in lignocellulosic hydrolysate (Supplementary Fig. S5). The expression of *dCas9-Mxi1* caused a high impairment on growth whereas strains expressing *dCas9* alone grew comparably to the control strain or better (Fig. 7a–d).

The growth of strains expressing *dCas9* from a low copy plasmid and sgRNAs targeting positions − 20, − 180 or − 380 relative to the TSS of *SSK2* (sg10, sg11 and sg12, respectively), was compared to strains expressing *dCas9* with the placeholder (Fig. 7a–d). The generation time of all strains expressing sgRNAs targeting *SSK2*p was reduced compared to the control strain (Fig. 7a). Moreover, the strain expressing sg10 showed a 2.3-fold increase in final biomass yield compared to the no sgRNA control (Fig. 7b, d). The lag phase of the strains was not affected by expression of any of the sgRNAs (Fig. 7c). When *SSK2* was disrupted from KE6-12, this modification led to an approx. 5 h longer lag phase compared to KE6-12, when growth in hydrolysate was evaluated (Fig. 7e).

## Discussion

In this study, we have successfully developed a CRISPRa/i toolkit for modulation of transcription in a polyploid, industrial yeast strain. The CRISPRa/i technology enables gradual modulation of gene expression allowing for screening of optimal expression levels. Regulation of transcription is a very sensitive balance and even small perturbations in gene expression can have great impact on the regulatory network and fitness of a cell subjected to stressful environments^46^.

We evaluated our CRISPRa/i toolkit by altering the expression of fluorescent proteins expressed under the strong *TDH3* promoter or under the relatively weak, endogenous *HRK1* promoter. An increased expression under *TDH3*p was observed for two out of six sgRNAs in strains expressing *dCas9-VPR* from a centromeric, low copy plasmid (Fig. 4e). In strains expressing *dCas9* alone or the *dCas9-Mxi* fusion two additional sgRNAs could decrease the expression of the fluorescent protein expressed under *TDH3*p (Fig. 4b, c). The most efficient sgRNAs, sg3 and sg4, both targeted a region of *TDH3*p close to a known upstream activating sequence, UAS2, −255 –309 bp relative to the TSS^47^. The highest repression of *TDH3*p was found in strains expressing sg2 targeting position − 127 (Fig. 4c). Our results suggest that a critical regulation element could be located in this region, since a repression of the promoter was obtained with all the plasmids tested, including *dCas9-VPR* (Fig. 4b–e). Despite designed to function as a transcriptional activator, *dCas9-VPR* has also previously in combination with certain sgRNAs been shown to confer transcriptional repression^32^.

Our previous work has shown that robustness and tolerance can vary greatly between industrial strains and evolved mutants^41,48^, highlighting the need for a system that can be rapidly implemented in novel hosts. The benefit of plasmid-based expression of dCas9 and sgRNA in comparison with genomic integration is that the CRISPRa/i toolbox can rapidly be introduced to new strains and for performing novel genomic regulation events. Targeting a new gene and phenotyping the alteration introduced can be achieved in a week.

A recent study, where a plasmid-based CRISPRa/i-system was developed demonstrated a threefold difference in butanediol production when the same regulation perturbations were implemented in two different laboratory yeast^49^. With our CRISPRa/i toolkit, a 2.5-fold change in gene expression was observed, when comparing the best performing sgRNA for repression using dCas9-Mxi1 with the most potent sgRNA for activation using dCas9-VPR. This fold-change in expression level is similar to what was previously seen in laboratory yeast strains^31,32,34^. Some previous studies report even stronger modulation of expression by CRISPRa/i, but the strength of developed CRISPRa/i systems has been shown to be largely dependent on the sgRNAs used (reviewed by Jensen^34^). Our study confirmed that current state-of-the-art software tools fail to predict how specific sgRNAs and scRNAs quantitatively interfere with the activity of a native promoter. The systematic evaluation of the efficiency of different sgRNAs was beyond the scope of this study and as the genome sequence of KE6-12 is unknown, sequence variability may have a caused further decrease in targeting efficiency when targeting native promoters as was previously reported by Smith et al.^30^. Furthermore, as previous work^30,32^ has shown that up to two thirds of the sgRNAs designed fail to function for CRISPRa/i, it may be that a higher effect of our CRISPRa/i toolkit would be possible by testing a larger set of sgRNAs.

In order to improve transformation efficiency and genetic stability, we chose to express the genes encoding the different dCas9 variants and the sgRNA from the same plasmid. The protospacer sequence of the sgRNA was co-transformed as a double stranded oligonucleotide and the correct assembly of the CRISPRa/i plasmids in all strains was confirmed by colony PCR (Supplementary Fig. S7), demonstrating that the homologous recombination capacity of our strain was remarkable. The design where the protospacer sequence is added as a double stranded oligonucleotide allows rapid targeting of any loci and circumvents the need for cloning when altering the expression of a new gene is desired. In fact, the phenotypes of the new strains can be characterized directly after transformation. Similarly, during strain construction by CRISPR/Cas9 based genome editing, a highly efficient single-step gene disruption in the polyploid industrial strain was obtained by using marker-free donor DNA with 40 bp homology to the integration site. When *Cas9* and the sgRNA were put on episomal plasmids, the plasmids could easily be cured from the yeast, effectively resulting in a marker-free, permanent modification.

High expression of *Cas9* has previously been reported to be toxic for industrial yeast^26^ and high expression of *dCas9* has been shown to be toxic for many bacteria (reviewed in^50^). Similarly, a few studies report that a high expression of the *VP16* in yeast led to a slower growth^51–53^. Here, we did not observe any significant growth defect with strains expressing *dCas9*, but we observed a growth defect when strains were expressing *dCas9-Mxi1* or *dCas9-VPR*, even without any sgRNA, (Fig. 2 and Supplementary Table S1). This effect was stronger when expressing the *dCas9* fusions from high copy plasmids compared to when low copy plasmids were used. Still, in order to achieve an increase in expression under *HRK1*p, a higher expression of *dCas9-VPR* and/or the sgRNA appeared to be needed. In contrast, the high copy variant of the CRISPRa/i plasmids did not outperform the low copy plasmids when targeting *TDH3*p, further demonstrating that the effect of CRISPRa/i is highly target-dependent, which emphasizes the need for a versatile CRISPRa/i toolkit. The most suited for plasmid of the toolkit may depend on the strain and target gene chosen.

The applicability of the developed CRISPRa/i toolkit was demonstrated by successfully improving the resistance of our industrial yeast strain towards lignocellulosic hydrolysate (Fig. 7). A number of previous studies report engineering of resistance to phenolics, furfural and carboxylic acids, the most common inhibitors found in lignocellulosic hydrolysates. Still, the sensitivity towards inhibitors in hydrolysates is a major bottleneck in developing robust strains^4^, therefore even small improvements in tolerance may have big effects on the profitability of biorefineries. The MAP kinase Ssk2 has been shown to be involved in furfural tolerance^35^ and it was also shown to be essential for combatting environmental stress^54^. In our study, *SSK2*p was targeted with dCas9, resulting in an improvement in growth in wheat straw hydrolysate (Fig. 7d). When *SSK2* was disrupted, growth was delayed (Fig. 7e), indicating that down-regulation of SSK2 is a better strategy compared to classical knockouts. This shows that the developed CRISPRa/i toolkit provides a rapid method for testing and validating gene targets for strain improvement. For industrial production strains it is likely beneficial to make the genetic targets identified using the CRISPRa/i toolkit stable, for instance through promoter substitution.

Collectively, this work demonstrates that CRISPRa/i can be used for transcriptional regulation in polyploid, industrial yeast strains. Moreover, this work has provided valuable information on transcriptional regulation using dCas9 and dCas9-fusions to transcriptional effectors. The CRISPRa/i plasmids have also been verified in laboratory CEN.PK strains (work in preparation), demonstrating that the toolkit can be expected to be widely applicable in different strain backgrounds. Even though there is still a need for further refining the toolkit, our results provide the framework for future work on CRISPRa/i based transcriptional modulation in polyploid yeast, accelerating the development of more robust strains to be used in biorefineries.

## Conclusions

The CRISPRa/i toolkit was shown to lead to transcriptional activation and repression in a polyploid yeast strain. The repression of a gene essential for environmental stress, *SSK2*, by CRISPRi demonstrated the potential of our toolkit for balancing and optimizing gene expression without genome editing. We anticipate that our CRISPRa/i toolkit can be widely used to accelerate the design-build-test cycle for developing various industrial production strains.

## Methods

### Plasmid assembly

All primers and plasmids used in this study are listed in the Supplementary material online. Primers and oligonucleotides used were ordered from Eurofins. Phusion High-Fidelity and Phire Hot Start II DNA polymerases were used for PCR amplification. All PCR fragments used for plasmid assembly were column purified (using the GeneJET PCR Purification Kit) and sequenced (Eurofins) prior to the final assembly. Chemically competent *Escherichia coli* DH5α cells were used for cloning and transformed cells were selected on LB with the appropriate antibiotics. All chemicals and reagents used were purchased from ThermoScientific™ unless otherwise noted.

CRISPRa/i plasmids were assembled using the MoClo Yeast Toolkit^55^ (Addgene #1000000061), which allows for rapid creation of plasmids with different selection cassettes or auxotrophic markers for expanding the versatility of the toolkit. New genetic parts containing *dCas9*, *Mxi1*, *VP64* or *VPR* where constructed by PCR amplification of the corresponding gene fragments from plasmids pTDH3-dCas9 (^36^, Addgene #46920) and pAG414GPD-dCas9-VPR (^38^ Addgene #63801). The parts were put into the entry plasmid pYTK001 using overhangs added to the amplification primers, resulting in plasmids EC0_1_dCas9, EC0_2_dCas9_VP64, EC0_3_dCas9_Mxi1 and EC0_4_dCas9_VPR. For the expression of the sgRNA, a placeholder sequence non-homologous to the yeast genome (primers hybridized by boiling followed by a gradual decrease in temperature) containing two BbsI sites was cloned into the plasmid #pYTK050 via BsmBI, resulting in the plasmid EC0_6_sgRNA, carrying a tRNA Phe promoter, the tSNR52 terminator and an HDV Ribozyme site. *dCas9* was expressed under the Sc*TEF1* promoter and Sc*TDH1* terminator. The final multigene plasmids, EC2_1_dCas9_sgRNA, EC2_2_dCas9_VP64_sgRNA, EC2_3_dCas9_Mxi1_sgRNA and EC2_4_dCas9_VPR_sgRNA carried *dCas9*, activator/repressor domains, the sgRNA scaffold and the CEN6/ARS4 origin of replication. High copy plasmids EC2_7_dCas9_HC_sgRNA and EC2_10_dCas9_VPR_HC_sgRNA were assembled with the 2µ origin of replication.

Linear oligonucleotides with 20 nt protospacers and 40 bp overlaps on either side of the sgRNA scaffold were assembled into the plasmids through in vivo homologous recombination. Prior to transformation, plasmids were digested by BpiI to remove the placeholder region in the sgRNA cassette. Clones were confirmed with colony PCR.

For gene editing by CRISPR/Cas9, the plasmid YN2_1_Cas9 was constructed assembling several genetic elements from the MoClo Yeast Toolkit, resulting in the *Cas9* gene from *Streptococcus pyrogens* under the Sc*PGK1* promoter and terminator, the tRNA Phe promoter, tSNR52 terminator, an HDV Ribozyme site for sgRNA expression and the sgRNA scaffold with a bacterial *GFP* expression cassette replacing the protospacer. The GFP expression cassette was amplified from pYTK050 using primers that added flanks compatible with the MoClo system. In plasmids YN2_1_Cas9_Ruby and YN2_1_Cas9_Venus for genome editing, the GFP cassette was removed by PstI digestion and replaced with the protospacer sequences.

A description of the parts assembled for each plasmid is provided in Supplementary Tables S4–S5.

### Strain construction and maintenance

The polyploid industrial *S. cerevisiae* strain KE6-12 was used as a parental strain. KE6-12 is derived from TMB400 by evolutionary engineering, with *XR* and *XDH* from *Pichia stipitis* integrated into the genome (Albers et al., unpublished). Yeast strains were transformed according to the Gietz method^56^ and cells were selected in YPD media supplemented with G418.

KE6-12 expressing *mRuby2* and *Venus* (KE6-12-Ruby and KE6-12-Ruby-Venus) were constructed by transformation of plasmids YN2_1_Cas9_Ruby or YN2_1_Cas9_Venus and a donor DNA containing the *mRuby2* or *Venus* expression cassettes (Supplementary Table S6). The fluorescence protein-encoding genes were amplified from EC1_10 or pYTK033, adding 40 bp homologous to the *PDR12* or *HRK1* locus.

Correct transformants, with p*TDH3*-*mRuby2*-t*TDH1* disrupting all the *PDR12* ORFs or placing *Venus* under the control of the native *HRK1* promoter at all chromosome copies, were selected. All the strains were verified by colony PCR (Supplementary Fig. S1,S3 and Table S7) and cured for plasmids before proceeding to transcriptional regulation using the CRISPRa/i toolkit or tolerance assays. Deletion of *PDR12* or *HRK1* was during non-stressful conditions previously shown not affect the growth of *S. cerevisiae*^9,57^. Strains were maintained in − 80 and the presence of plasmids expressing *dCas9* was confirmed in all the strains at the end of the growth in hydrolysate (Supplementary Fig. S8).

KE6-12 strain with *SSK2* disrupted was constructed by co-transformation of BpiI-digested EC2_5 plasmid (Supplementary Table S5) and a donor DNA containing a barcode sequence with flanking 40 bp homologous arms to *SSK2* locus (Supplementary Table S6). Correct transformants with all the *SSK2* ORFs disrupted were selected by colony PCR (Supplementary Fig. S6).

### sgRNA design

The yeast CRISPRi webtool^30^ and CRISPR-ERA^58^ were used to design the sgRNAs, using as a criteria ATAC-seq values around 1 and nucleosome-free areas, to ensure accessibility to the target genes (Supplementary Table S7). Additionally, poly-N and presence of off-targets were avoided and GC content between 30 and 55 was preferred. For KE6-12-Ruby, positions at + 1, − 127, − 277, − 351, − 469 or − 541 bp relative to the TSS were chosen to cover most of the *TDH3* promoter region. A spacing of around 200 base pairs was chosen to cover most of the promoter region. For KE6-12-Ruby-Venus, 3 sgRNAs were designed targeting *HRK1*p at − 41, − 213 or − 355 bp relative to the TSS, while for *SSK2*p positions targeting regions at − 20, − 180 or − 380 bp relative to the TSS were chosen. Positions for sgRNAs targeting *BDH2*p or *ISC1*p can be found in Supplementary Table S8. Verification of sgRNA integration into the expression plasmid was done by colony PCR for strains with sgRNAs targeting *TDH3*p and *HRK1*p (Supplementary Table S9). Oligonucleotides used as placeholder or sgRNA are provided in the Supplementary Table S8.

### Media

The *E. coli* strain was grown in liquid or solid (supplemented with 15 g/L agar) Luria–Bertani (LB) medium (10 g/L bacto tryptone, 5 g/L yeast extract, 10 g/L NaCl) containing 25 mg/L chloramphenicol, 100 mg/L ampicillin or 50 mg/L neomycin. Yeast strains were grown in liquid or solid (supplemented with 15 g/L agar) yeast extract peptone dextrose (YPD) medium (10 g/L yeast extract, 20 g/L peptone, and 20 g/L glucose). Strains containing plasmids were grown on YPD medium supplemented with 200 mg/L geneticin. For the tolerance assays in minimal media, YNB media (1.9 g/L YNB, 0.79 g/L CSM and 5 g/L ammonium sulphate, 20 g/L glucose) containing geneticin (200 mg/L) for CRISPRa/i strains and supplemented with either acetic acid (100 mM) furfural (20 mM) or vanillin (12 mM) was used. The medium was adjusted at pH 4.5 and buffered with succinate (50 mM). For the tolerance assay in hydrolysates, wheat straw hydrolysate with a composition of 68.8 g/L glucose, 36.4 g/L xylose, 1.2 g/L formic acid, 4.7 g/L acetic acid, 0.6 g/L 5-(hydroxymethyl)furfural (HMF), and 3.0 g/L furfural^45^ was used. The hydrolysate was kindly provided by Dr M. Galbe at Lund University (Sweden).

### Culture conditions

For growth characterization and flow cytometry analysis, transformants carrying CRISPRa/i plasmids were grown in liquid medium using the Growth Profiler 960 platform (Enzyscreen). Precultures were inoculated from a fresh colony and incubated overnight at 30 °C and 200 rpm in YPD, and then transferred into 96-well, transparent bottom, white-walled plates (CR1496d, EnzyScreen) with 250 µL of YPD at an initial OD_600_ of 0.05. Cultures were incubated at 30 °C and 250 rpm for 24 h. OD_600_ values were analyzed using the PRECOG software^59^ to extract the length of the lag phase, the generation time and the biomass (OD_600_) yield. The average and standard deviation of three biological replicates are reported.

For real-time monitoring of mRuby2 fluorescence and growth assessment of KE6-12 in wheat straw hydrolysate, strains were grown in microbioreactors, using the Biolector platform (m2p-Laboratories GmbH). Precultures were inoculated from a fresh colony and incubated overnight at 30 °C and 200 rpm in YPD medium in a 96-well microtiter plate. For KE6-12-Ruby strains, overnight cultures were transferred into a 48-well microtiter plate (48-B FlowerPlate, m2p-labs) with 1 mL of YPD at an initial OD_600_ of 0.05. For KE6-12, precultures were centrifuged and resuspended in sterile water to a final OD_600_ of 5 and 100 µL of cell suspension was transferred into the 48-well microtiter plate containing 900 µL of wheat straw hydrolysate (diluted to 70%). All cultures were incubated at 30 °C, 1200 rpm, at a humidity of 85%. On-line parameters were measured in 20 min intervals, using an excitation and emission filter at 600 nm for scattered light, with a filter gain of 10 and a filter gain of 20 for YPD and hydrolysate media, respectively. mRuby2 fluorescence was measured at 580/610 nm and a gain of 100.

### Flow cytometry measurements

For fluorescence measurements, transformants carrying CRISPRa/i plasmids were grown for 24 h using the Growth Profiler, in 96-well microtiter plates containing 250 µL of YPD, incubated at 30 °C and 250 rpm. Fluorescence was analyzed by flow cytometry using the Guava easyCyte 8HT system (Merck Millipore). All samples were, prior to analysis by flow cytometry, diluted in sterile water to a final concentration of 200–500 cells/µL and a total amount of 5,000 cells were measured per analysis at a flow rate of 0.59 mL/s. An excitation wavelength of 488 nm and a detection wavelength of 583 nm and 525 nm were used for KE6-12-Ruby and KE6-12-Ruby-Venus, respectively. The average and standard deviation of the geometric mean for three biological and three technical replicates is reported.

### Statistical analysis

Pairwise comparisons were carried out with R, using the unpaired Student’s T-test for normally distributed data. The normal distribution of the data was determined using the Shapiro–Wilk test. Statistical relevance due to the p-value (P) obtained by the statistical tests performed is shown with “ns” for *p* > 0.05, “*” for *p* ≤ 0.05, “**” for *p* ≤ 0.01, “***” for *p* ≤ 0.001 and “****” for *p* ≤ 0.0001.

## Supplementary information

Supplementary information

## Data Availability

All data generated or analyzed during this study are included in this published article and its supplementary files. Plasmids generated will be publicly available through Addgene.
