# Supplementary material for: A CRISPR activation and interference toolkit for industrial Saccharomyces cerevisiae strain KE6-12
Source: Sci Rep. 2020 Sep 3;10:14605. doi: 10.1038/s41598-020-71648-w (PMC7471924; doi:10.1038/s41598-020-71648-w)
Supplement: Supplementary file 1 — Supplementary information [file 41598_2020_71648_MOESM1_ESM.pdf]

Supplementary material

A CRISPR activation and interference toolkit for industrial *Saccharomyces cerevisiae* strain KE6-12

Elena Cámara, Ibai Lenitz, Yvonne Nygård

Figure S1: Verification of *mRuby2* integration and expression in KE6-12

Figure S2: Growth curves of KE6-12-Ruby strains expressing CRISPRa/i plasmids

Figure S3: Verification of *Venus* integration and expression in KE6-12-Ruby

Figure S4. Growth curves of KE6-12 strains expressing dCas9 or dCas9-VPR and sgRNAs targeting different promoters

Figure S5. Growth curves of KE6-12 strains expressing CRISPRi plasmids

Figure S6. Verification of *SSK2* disruption in KE6-12

Figure S7. PCR verification of sgRNA integration into the CRISPRa/i plasmids

Figure S8. PCR verification of KE6-12 clones after growth in hydrolysate

Table S1. Growth of KE6-12-Ruby carrying different CRISPRa/i plasmids

Table S2. mRuby2 fluorescence of KE6-12-Ruby strains

Table S3. Venus fluorescence of KE6-12-Ruby-Venus strains

Table S4: Primers used for amplification of PCR products for cloning

Table S5: MoClo plasmid construction

Table S6: Primers used for donor DNA amplification or creation

Table S7: Scores obtained by CRISP-ERA and YEAST-CRISPRi for the sgRNAs selected

Table S8. Oligonucleotides used as placeholder or sgRNA

Table S9: Primers used for verification of sgRNA integration and strain construction

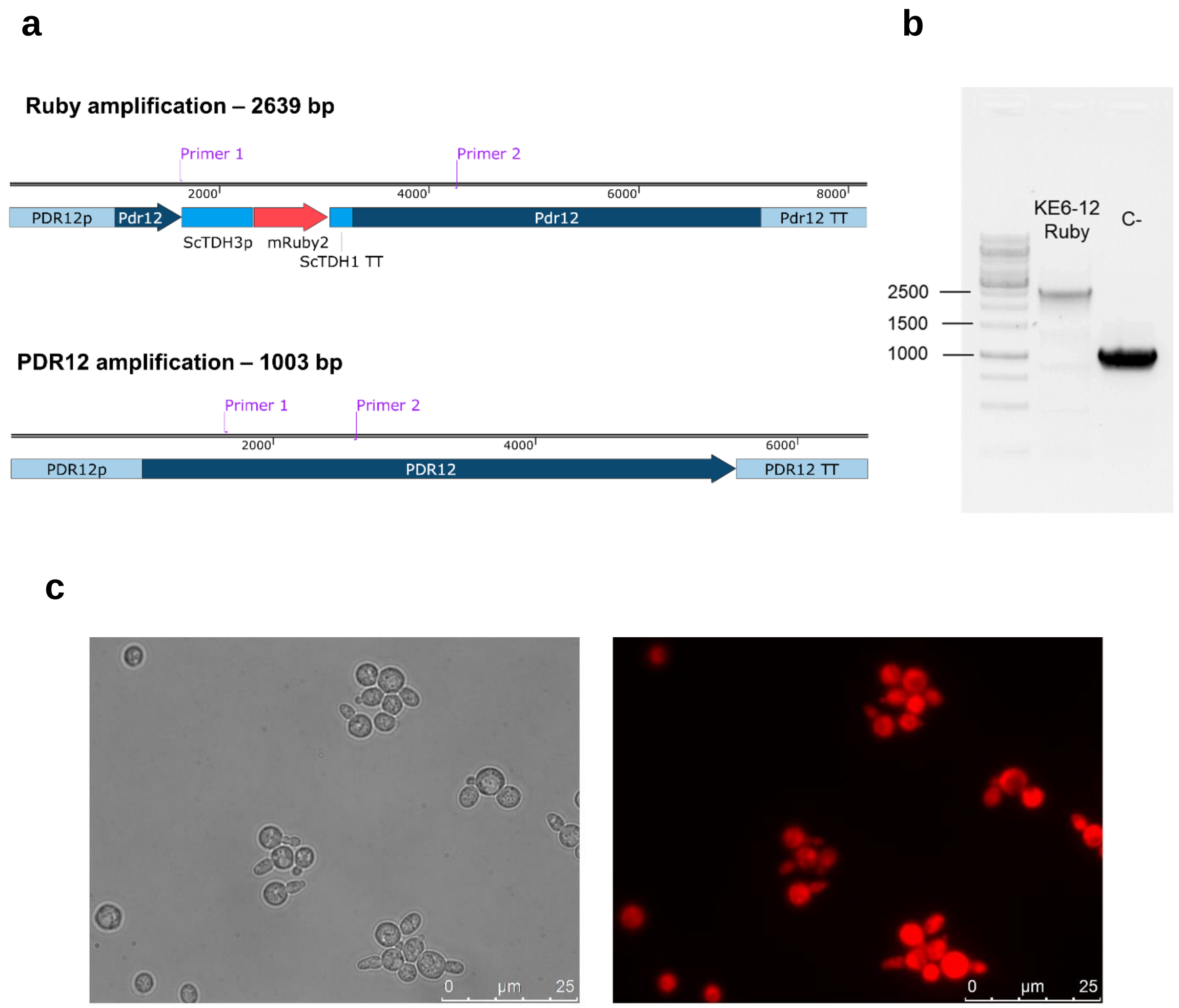

Figure S1. Verification of *mRuby2* integration and expression in KE6-12. Primers used specified in Table S7. a) PCR amplification with primer 1 and 2 results in a fragment of 2639 bp when *PDR12* is correctly disrupted by the integration of the *mRuby* cassette. Amplification of the intact *PDR12* gene would result in a fragment of 1003 bp. b) Verification of *mRuby2* integration in the KE6-12. As a negative control (C-) PDR12 was amplified from a KE6-12 strain. The GeneRuler 1 kb DNA Ladder ready-to-use (Thermo Scientific) was used for size quantification. c) mRuby2 fluorescence expression of the verified strain, named KE6-12-Ruby. Visualization was done with a Leica DMI4000B microscope, using the RFP filter, exposure time of 30ms and gain of 1.5. Magnification Camera 70x and Visual 1000x.

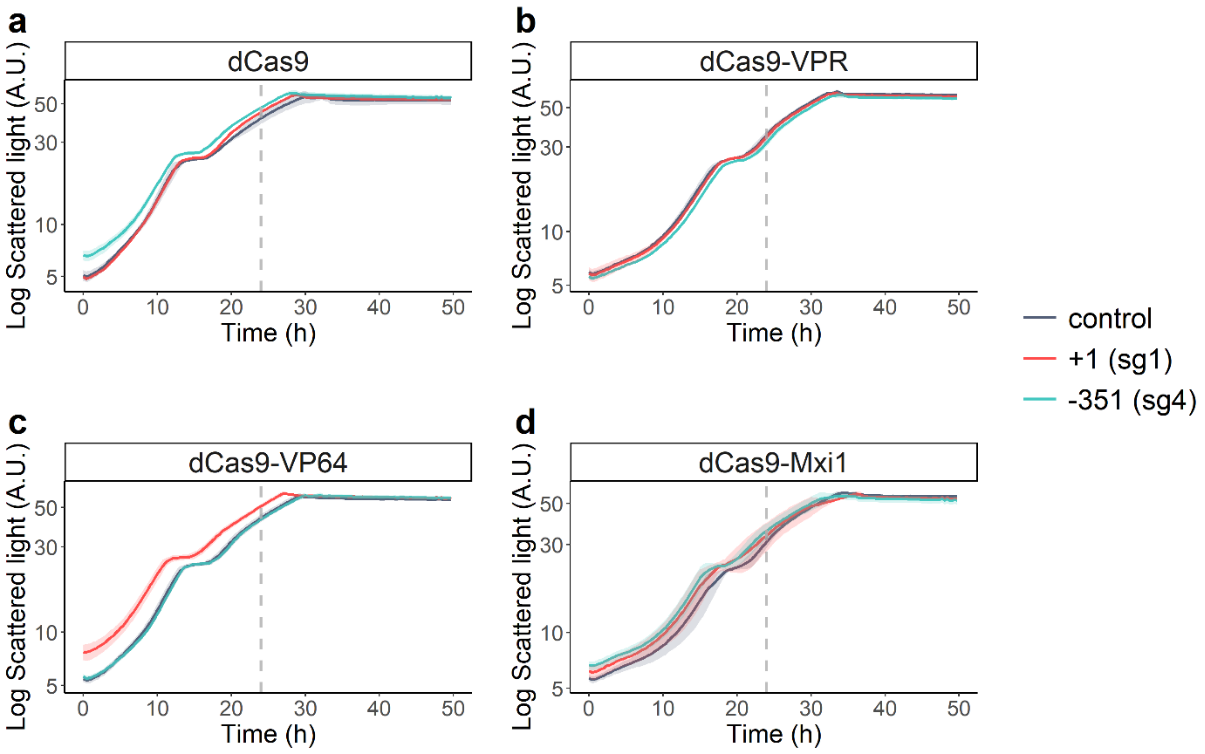

Figure S2. Growth curves of KE6-12-Ruby strains expressing CRISPRa/i plasmids, measured as biomass light scattering. dCas9 was targeting a region at +1 (sg1; red line) or -351 (sg4; turquoise line) bp from the transcription starting site, the control strain carried the CRISPRa/i plasmid with the placeholder (control; grey line). Samples for flow cytometry were taken after 24 hours of cultivation (dashed line). Data obtained from three biological replicates monitored with a Biolector; shadowed regions show the standard deviation (SD).

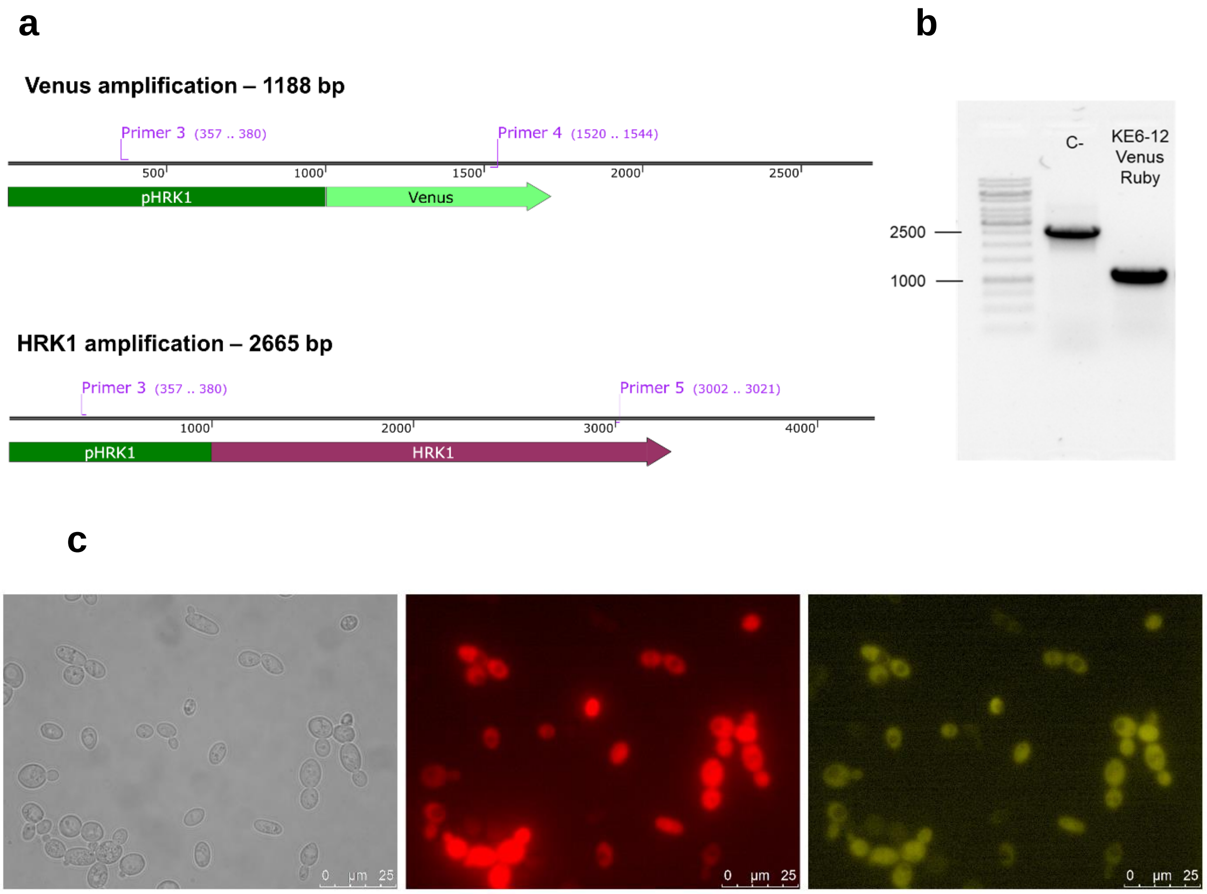

Figure S3. Verification of *Venus* integration and expression in KE6-12-Ruby. Primers used specified in Table S7. a) The expected size of the amplification product was 1188 bp when *Venus* was correctly integrated in the *HRK1* locus, or 2665 bp when *HRK1* was intact. b) Verification of *Venus* integration in the KE6-12-Ruby strain. As a negative control (C-) HRK1 was amplified from a KE6-12-Ruby strain. The GeneRuler 1 kb DNA Ladder ready-to-use (Thermo Scientific) was used for size quantification. c) mRuby2 (center) and Venus (right) fluorescence of the verified strain, named KE6-12-Ruby-Venus. The visualization was done using a Leica DMI4000B microscope, using the RFP and the GFP filter for mRuby2 and Venus fluorescence, respectively. The exposure time was 30ms and gain of 1.5. Magnification Camera 70x and Visual 1000x.

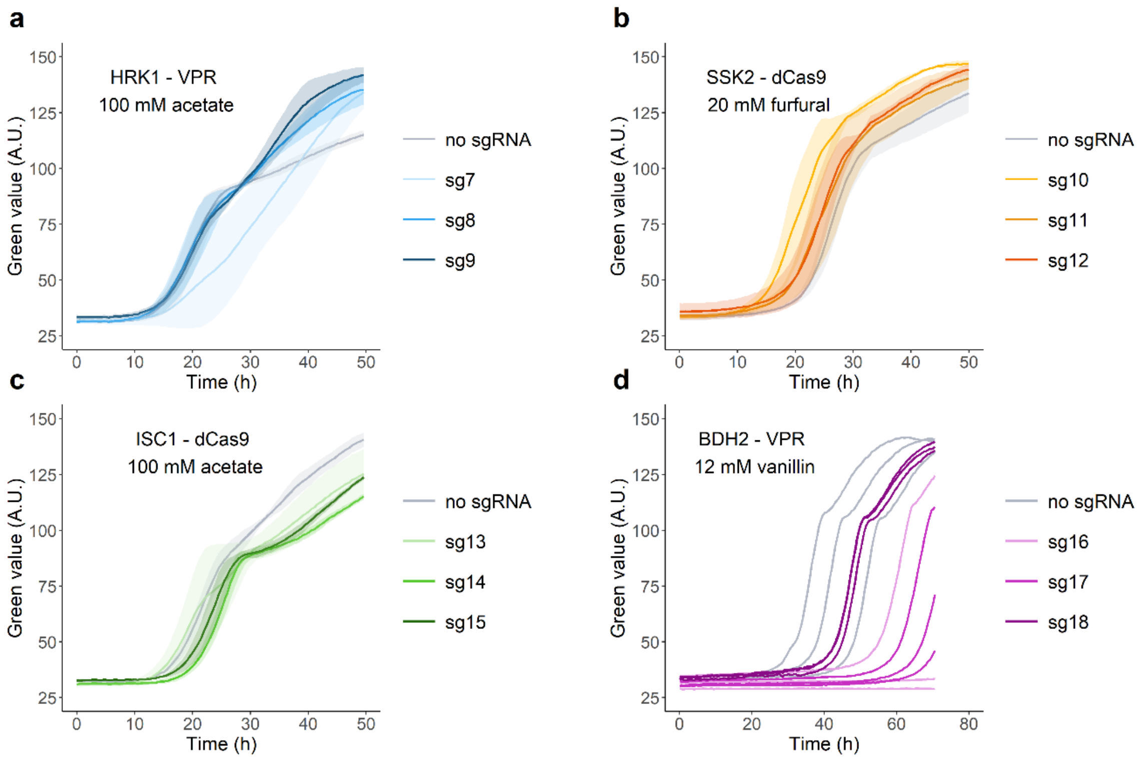

Figure S4. Growth curves of KE6-12 strains in minimal medium buffered with different inhibitors expressing a CRISPRa/i plasmid and an sgRNA targeting promoters of a) *HRK1*, b) *SSK2*, c) *ISC1* and d) *BDH2* or the placeholder (no sgRNA strain). Data obtained from three biological replicates monitored with a Growth Profiler; shadowed regions show the standard deviation (SD).

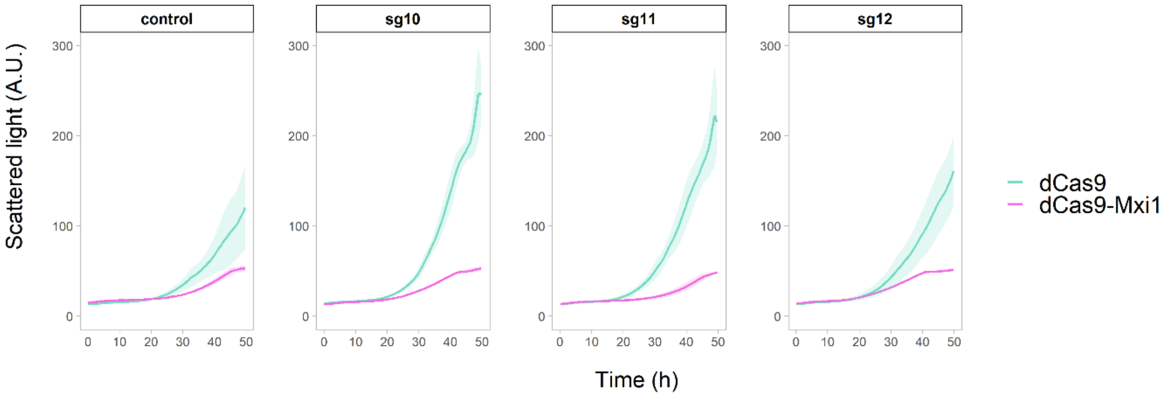

Figure S5. Growth curves of KE6-12 strains expressing CRISPRi plasmids, measured as biomass light scattering. The strains express *dCas9* (turquoise line) or *dCas9-Mxi1* (purple line) and an sgRNA targeting *SSK2*p. Data obtained from three biological replicates monitored with a Biolector; shadowed regions show the standard deviation (SD).

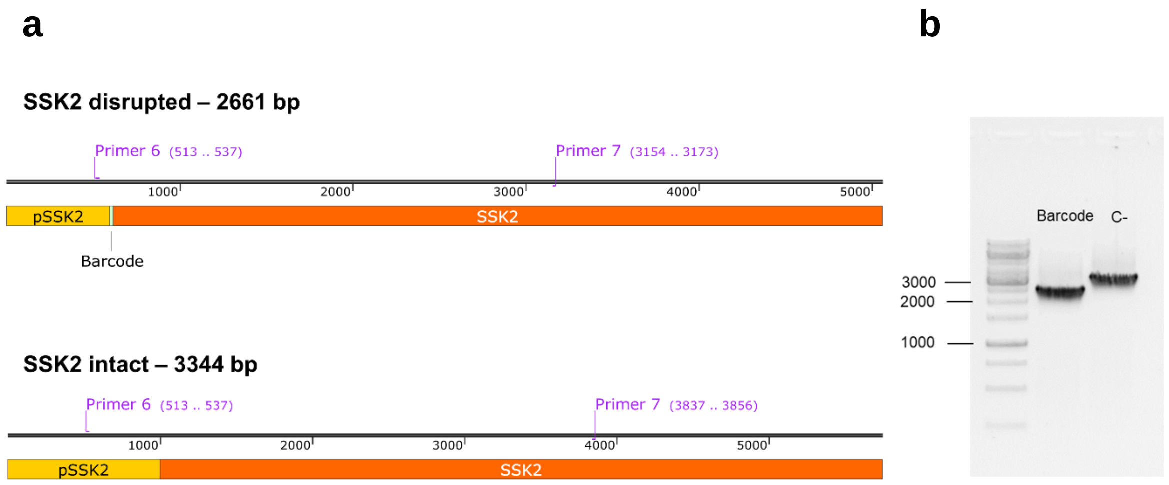

Figure S6. Verification of *SSK2* disruption. a) Disruption of *SSK2* was done by insertion of a barcode with a stop codon and a removal of 683 bp. The expected size of the amplification product was 2661 bp when *SSK2* was correctly disrupted, or 3344 bp when *SSK2* was intact. b) Verification of *SSK2* disruption in the KE6-12 strain. As a negative control (C-) *SSK2* was amplified from KE6-12. The GeneRuler 1 kb DNA Ladder ready-to-use (Thermo Scientific) was used for size quantification. Primers used specified in Table S7.

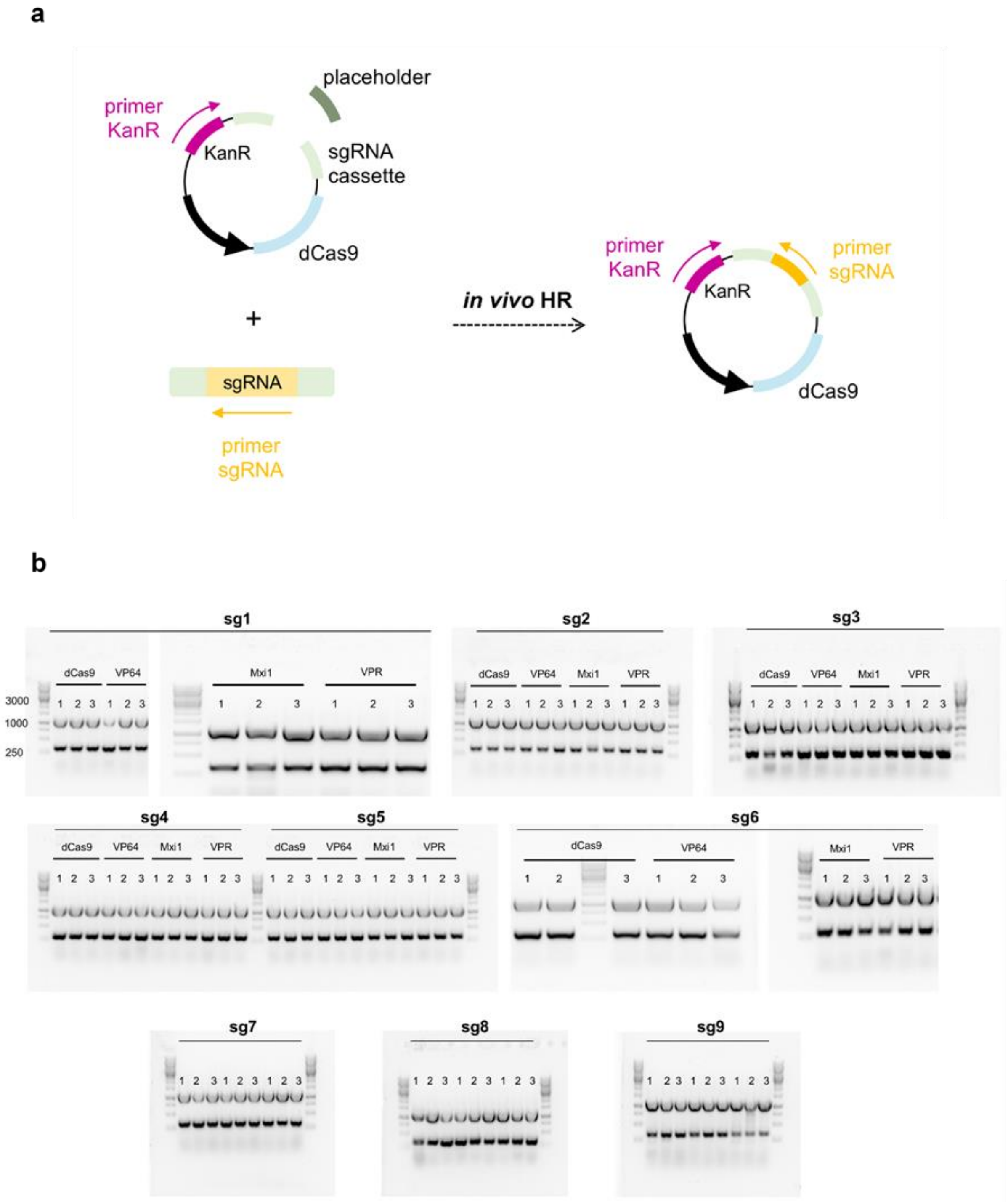

Figure S7. Colony PCR verification of sgRNA integration into the CRISPRa/i plasmids. a) The target-specific sgRNA from a double stranded oligonucleotide was inserted into the CRISPRa/i plasmid through yeast *in vivo* homologous recombination. Primers amplifying a fragment from the resistance marker to the specific sgRNA sequence of each construct were used to verify the assembly. b) The expected size of the product (from the sgRNA to p*TEF* of KanR) was 350 bp (lower band). As an amplification control for the colony PCR, a set of primers for *ADE2* amplification were added (1250 bp product, upper band). GeneRuler 1 kb DNA Ladder ready-to-use (Thermo Scientific) used for size quantification.

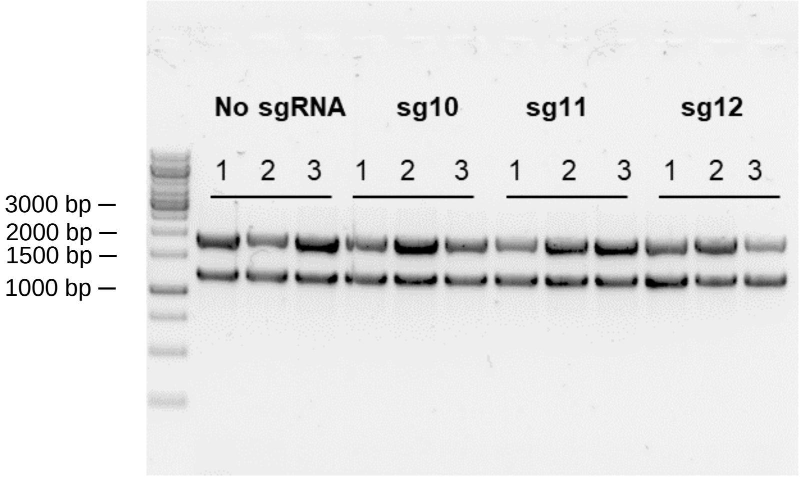

Figure S8. PCR verification of KE6-12 clones after growth in hydrolysate. A fragment of *dCas9* was amplified in all the clones to verify the presence of the CRISPRi plasmid; the expected size was 1955 bp (upper band). As an amplification control for the colony PCR, a set of primers for *ADE2* amplification were added (1250 bp product, lower band). GeneRuler 1 kb DNA Ladder ready-to-use (Thermo Scientific) used for size quantification.

Table S1. Growth of KE6-12-Ruby expressing different CRISPRa/i plasmids. The growth parameters were calculated using PRECOG (Fernandez-Ricaud et al. 2016, BMC Bioinformatics 2016;17:1–15). The No_CRISPRa/i strain was generated by transforming KE6-12-Ruby with an empty plasmid lacking dCas9 or sgRNA expression cassettes. Strains were grown in microbioreactors in YPD medium for 35 h. Data represents three biological replicates ± SD. Statistical analysis was performed using the Student's t-test. A P-value lower than 0.05 was considered statistically significant. Statistical relevance obtained by the statistical tests performed is shown with "ns" for $p > 0.05$, "*" for $p \leq 0.05$, "**" for $p \leq 0.01$, "***" for $p \leq 0.001$ and "****" for $p \leq 0.0001$.

	**Lag time (h)**	**Sign. level**	**Gen time (h)**	**Sign . level**	**Yield (∆Scat. light)**	**Sign. level**
No_CRISPRa/i	3.34 ± 0.07	-	2.93 ± 0.05	-	50.80 ± 0.06	-
dCas9	4.56 ± 0.36	*	3.81 ± 0.31	ns	49.79 ± 4.23	ns
dCas9-VP64	4.88 ± 0.22	**	4.03 ± 0.06	****	52.83 ± 0.91	ns
dCas9-Mxi1	9.13 ± 1.45	*	4.64 ± 0.01	***	42.08 ± 1.34	*
dCas9-VPR	7.52 ± 0.77	*	4.83 ± 0.13	***	47.99 ± 1.67	ns
dCas9-HC	5.49 ± 1.86	ns	5.00 ± 0.04	****	42.07 ± 2.86	*
dCas9-VPR-HC	16.80 ± 0.60	***	6.30 ± 0.29	**	17.22 ± 0.28	****

Table S2. mRuby2 fluorescence of KE6-12-Ruby strains. The strains carry different CRISPRa/i plasmids with different sgRNAs or the sgRNA placeholder (No sgRNA). % increase refers to the fluorescence change compared to the No sgRNA control. Fluorescence expressed as geometric mean. Data represents three biological and three technical replicates, measured by flow cytometry (samples taken after 24h of culture).

	**Strain**	**MFI**	**% increase**		**Strain**	**MFI**	**% increase**
**dCas9**	No sgRNA	22.7	-	**dCas9-VPR**	No sgRNA	18.1	-
	sg1	21.4	-5.4		sg1	17.5	-3.0
	sg2	14.6	-35.5		sg2	16.6	-8.3
	sg3	17.2	-24.0		sg3	28.3	56.6
	sg4	17.3	-23.7		sg4	29.8	64.7
	sg5	16.6	-26.8		sg5	20.8	14.9
	sg6	22.7	-0.1		sg6	19.5	8.2
**dCas9-VP64**	No sgRNA	21.5	-	**dCas9-HC**	No sgRNA	18.8	-
	sg1	22.1	2.7		sg1	19.5	3.9
	sg2	16.2	-24.8		sg2	13.7	-27.0
	sg3	23.8	10.6		sg3	16.2	-13.8
	sg4	24.1	12.3		sg4	16.7	-11.1
	sg5	19.1	-11.1		sg5	15.4	-18.2
	sg6	21.5	0.0		sg6	19.1	1.5
**dCas9-Mxi1**	No sgRNA	21.1	-	**dCas9-VPR-HC**	No sgRNA	20.6	-
	sg1	22.8	7.6		sg1	21.8	5.6
	sg2	11.7	-44.7		sg2	18.6	-9.9
	sg3	12.3	-42.1		sg3	24.8	20.5
	sg4	14.3	-32.3		sg4	25.0	21.2
	sg5	14.3	-32.5		sg5	20.0	-2.9
	sg6	22.5	6.3		sg6	22.5	9.1

Table S3. Venus fluorescence of KE6-12-Ruby-Venus strains. The strains carry different CRISPRa/i plasmids with different sgRNAs or the sgRNA placeholder (No sgRNA). % increase refers to the fluorescence change compared to the No sgRNA control. Fluorescence expressed as geometric mean. Data represents three biological and three technical replicates, measured by flow cytometry.

Plasmid	Strain	MFI	% increase
**dCas9**	No sgRNA	89.8	-
	sg7	88.7	-1.2
	sg8	89.1	-0.8
	sg9	97.5	8.6
**dCas9-HC**	No sgRNA	72.7	-
	sg7	69.9	-3.9
	sg8	73.6	1.2
	sg9	76.8	5.7
**dCas9-Mxi1**	No sgRNA	86.9	-
	sg7	77.4	-10.9
	sg8	84.6	-2.6
	sg9	64.5	-25.8
**dCas9-VPR-HC**	No sgRNA	56.8	-
	sg7	73.2	28.9
	sg8	76.3	34.2
	sg9	88.7	56.2
**dCas9-VPR**	No sgRNA	79.9	-
	sg7	77.9	-2.6
	sg8	81.8	2.3
	sg9	101.8	27.3

Table S4. Primers used for amplification of PCR products for cloning. All plasmids were verified by sequencing.

**Plasmid ID**	Description	Primer sequences (5'->3')	Template
EC0_1	Entry plasmid pYTK001 carrying SV40 NLS - dCas9 - SV40 NLS	F1:gatccgtctcatcggtctcatatgccaaagaagaagagaaaggtagg<br>R1:tcgtctctttcccctgaatcaaagagga<br>F2:tcgtctctggaaaccgctgaagcaacccg<br>R2:atgccgtctcaggtctcaggatCCtaccttgcgctttttcttgggatcccctccg	pTDH3-dCas9-Mxi1 (Addgene #46921)
EC0_2	Entry plasmid pYTK001 carrying VP64 - SV40 NLS	F:gatccgtctcatcggtctcaatccgacgcattggacgatt<br>R:atgccgtctcaggtctcagccactcgagttaaactttgcgtttctttttcggaatcagcatgtc	pAG414GPD-dCas9-VPR (Addgene #63801)
EC0_3	Entry plasmid pYTK001 carrying Mxi1-SV40 NLS	F:gatccgtctcatcggtctcaatccatggaacgtgtgagaa<br>R:atgccgtctcaggtctcagccactcgagttaaactttgcgtttctttttcggtctgggagagggcatgctagg	pTDH3-dCas9-Mxi1 (Addgene #46921)
EC0_4	Entry plasmid pYTK001 carrying VPR (VP64 - SV40 NLS - p65 - Rta)	F1:gatccgtctcatcggtctcaatccgacgcattggacgatt<br>R1: acgtctctttcttcatcgggatcct<br>F2:acgtctctagaaacgagccaggctgtca<br>R2:atgccgtctcaggtctcagccactcgagttaaaacagagatgtgtcgaaga	pAG414GPD-dCas9-VPR (Addgene #63801)

Table S5. MoClo plasmid construction. Plasmids were constructed using PCR products, dsDNA fragments and MoClo compatible plasmids.

Plasmid ID	Description	Parts assembled
Level 1		
EC1_1	Plasmid for expressing *dCas9*	pYTK002 / pYTK013 / EC0_1 / pYTK056 / pYTK067 / pYTK077 / pYTK081 / pYTK083
EC1_2	Plasmid for expressing *dCas9* and *VP64*	pYTK002 / pYTK013 / EC0_1 /EC0_2 / pYTK066 / pYTK067 / pYTK077 / pYTK081 / pYTK083
EC1_3	Plasmid for expressing *dCas9* and *Mxi1*	pYTK002 / pYTK013 / EC0_1 /EC0_3 / pYTK066 / pYTK067 / pYTK077 / pYTK081 / pYTK083
EC1_4	Plasmid expressing *dCas9* and *VPR*	pYTK002 / pYTK013 / EC0_1 /EC0_4 / pYTK066 / pYTK067 / pYTK077 / pYTK081 / pYTK083
EC1_7	Plasmid expressing sgRNA cassette and the placeholder	pYTK003 / EC0_6 / pYTK072 / pYTK077 / pYTK081 / pYTK083
EC1_8	Plasmid expressing *Cas9*	pYTK002 / pYTK013 / pYTK036 / pYTK056 / pYTK067 / pYTK077 / pYTK081 / pYTK083
EC1_9	Plasmid backbone for level 2 assembly of low copy plasmids	pYTK008 / pYTK047 / pYTK073 / pYTK077 / pYTK081 / pYTK084
EC1_10	Plasmid expressing *mRub*y2 under *TDH3*p	pYTK002 / pYTK009 / pYTK034 / pYTK056 / pYTK072 / pYTK077 / pYTK081 / pYTK083
EC1_11	Plasmid backbone for level 2 assembly of multi-copy plasmids	pYTK008 / pYTK047 / pYTK073 / pYTK077 / pYTK082 / pYTK084
YN1_1	Plasmid expressing *Cas9*	pYTK002 / pYTK011 / pYTK036 / pYTK054 / pYTK072 / pYTK077 / pYTK081 / pYTK083
YN1_2	Plasmid backbone for YN level 2 plasmid	pYTK002 / pYTK073 / pYTK077 / pYTK081 / pYTK082
Level 2		
EC2_1	Low copy plasmid with *dCas9* and sgRNA expression cassettes	EC1_1 / EC1_7 / EC1_9
EC2_2	Low copy plasmid with *dCas9-VP64* and sgRNA expression cassettes	EC1_2 / EC1_7 / EC1_9
EC2_3	Low copy plasmid with *dCas9-Mxi1* and sgRNA expression cassettes	EC1_3 / EC1_7 / EC1_9
EC2_4	Low copy plasmid with *dCas9- VPR* and sgRNA expression cassettes	EC1_4 / EC1_7 / EC1_9
EC2_5	Plasmid for genome editing by CRISPR/Cas9	EC1_7 / EC1_8 / EC1_9
EC2_7	High copy plasmid with *dCas9* and sgRNA expression cassettes	EC1_1 / EC1_7 / EC1_11

EC2_10	High copy plasmid expressing dCas9, VPR and the sgRNA expression cassette	EC1_4 / EC1_7 / EC1_11
YN2_1	Plasmid for genome editing by CRISPR/Cas9	YN1_1 / YN1_2
YN2_1_Cas9_Ruby	CRISPR/Cas9 plasmid expressing sgRNA targeting PDR12 locus for mRuby2 insertion	YN2_1 + sg_PDR12
YN2_1_Cas9_Venus	CRISPR/Cas9 plasmid expressing sgRNA targeting *HRK1* locus for Venus insertion	YN2_1 + sg_HRK1

Table S6. Primers used for donor DNA amplification or creation.

**Description**	**Primer sequences (5'->3')**	**Template**
Primer set for amplification of *mRuby2* with homology arms to *PDR12* locus	F:atgtacaaggtgaattctcctatgatggtctggaccaaagcagttcgagtttatcattatcaatactgccatttc<br>R:tgggaaatggaaatcaagctcgggacagtaaataacgtaacgttcagggtaatatattttaaccgccg	EC1_10
Primer set for amplification of *mRuby2* with homology arms to *HRK1* locus	F:taatacggtagaactatttctcgtataaagatgtctaaaggtgaagaattattcactggtgt<br>R:aaagaagtgaaaataattgagtagttcgtatcaggatcctttgtacaattcatccatacca	pYTK033
SSK2_barcode (ds oligo used for *SSK2* disruption)	F:cctgaaacagtgggtattttagcagttttgcattattaatggccaggcggtaattaggtaaatgccatttctgggtcctccagctcctcggcaagatcta<br>R:tagatcttgccgaggagctggaggacccagaaatggcatttacctaattaccgcctggccattaataatgcaaaactgctaaaatacccactgtttcagg	

Table S7. Scores obtained by CRISP-ERA (Liu et al. Bioinformatics. 2015;31:3676–8) and YEAST-CRISPRi (Smith et al. Genome Biol. 2016;17:45) the sgRNAs selected.

			CRISP-ERA			YEAST-CRISPRi	
**ID**	**POS. (rel. to TSS)**	**SEQUENCE**	**STRAND**	**E-SCORE**	**OFFTARGET**	**NUCLEOSOME**	**CHROMATINE**
sg1	0	ACACACATAAACAAACAAAA	+	15	0	-	-
sg2	-127	AATAAGTATATAAAGACGGT	+	15	0	0.03	0.35
sg3	-277	CTGGAGTAAATGATGACACA	+	20	0	0.04	0.82
sg4	-351	TAGCGCAACTACAGAGAACA	+	20	0	0.11	0.87
sg5	-469	CTGGCATCCACTAAATATAA	+	20	0	0.13	0.61
sg6	-541	TACATGCCCAAAATAGGGGG	+	20	0	0	0.59
sg7	-41	AAGTGGAAAACTGTTAAATC	-	20	0	0.04	0.37
sg8	-213	TAAGCGGTTGGGAACTGTCT	-	15	0	0.3	0.24
sg9	-355	AAGGGGCGGCGACGACGTAG	-	15	0	0	0.59
sg10	-20	CCAAATCTGCAGATTAGAAG	+	20	-19	0.51	0.1
sg11	-183	CGTTTCATACCCTGAAACAG	+	20	-16	0.27	0.33
sg12	-320	TCGTCCAATTCCATCTCTAC	+	20	-16	0.32	0.41
sg13	-32	TCATTCTTGAACCTTGTCAT	-	20	-48	0.18	0.09
sg14	-177	AGCAAGGGGGTTAACCACCA	-	20	-48	0.74	0.16
sg15	-321	AGAAACTGAAATCCCTTCCG	-	15	-48	0	0.36
sg16	+24	GTTCACGAGAGGAAGGAAGA	-	20	0	0.12	0.33
sg17	-143	ATGAGCGGTTTTAATTGAAT	-	15	0	0.31	0.54
sg18	-260	TTTGCTGACGTGCCAGTACC	-	20	0	0.37	0.37

Table S8. Oligonucleotides used as placeholder or sgRNA. In capital letters, the 20-nt specific sgRNA protospacer sequence. TSS = Transcription start site

dsDNA ID	Description	Oligonucleotide sequences (5'->3')
Place-holder	sequence non-homologous to the yeast genome containing two BbsI sites for Moclo cloning	F: gactcgctatcgtcttctcacgaagacttactca<br>R: aaactgagtaagtcttcgtgagaagacgatagcg
sg1	sgRNA targeting *TDH3*p at +1 relative to the TSS	F:ggcgccggctgggcaacaccttcgggtggcgaatgggactACACACATAA ACAAACAAAAgttttagagctagaaatagcaagttaaaataaggctagtc<br>R:gactagccttattttaacttgctatttctagctctaaaacTTTTGTTTGTTTATG TGTGTagtcccattcgccacccgaaggtgttgcccagccggcgcc
sg2	sgRNA targeting at -127 relative to the TSS	F:ggcgccggctgggcaacaccttcgggtggcgaatgggactAATAAGTATAT AAAGACGGTgttttagagctagaaatagcaagttaaaataaggctagtc<br>R:gactagccttattttaacttgctatttctagctctaaaacACCGTCTTTATATA CTTATTagtcccattcgccacccgaaggtgttgcccagccggcgcc
sg3	sgRNA targeting at -277 relative to the TSS	F:ggcgccggctgggcaacaccttcgggtggcgaatgggactCTGGAGTAAA TGATGACACAgttttagagctagaaatagcaagttaaaataaggctagtc<br>R:gactagccttattttaacttgctatttctagctctaaaacTGTGTCATCATTTA CTCCAGagtcccattcgccacccgaaggtgttgcccagccggcgcc
sg4	sgRNA targeting *TDH3*p at -351 relative to the TSS	F:ggcgccggctgggcaacaccttcgggtggcgaatgggactTAGCGCAACT ACAGAGAACAgttttagagctagaaatagcaagttaaaataaggctagtc<br>R:gactagccttattttaacttgctatttctagctctaaaacTGTTCTCTGTAGTT GCGCTAagtcccattcgccacccgaaggtgttgcccagccggcgcc
sg5	sgRNA targeting *TDH3*p at -469 relative to the TSS	F:ggcgccggctgggcaacaccttcgggtggcgaatgggactCTGGCATCCA CTAAATATAAgttttagagctagaaatagcaagttaaaataaggctagtc<br>R:gactagccttattttaacttgctatttctagctctaaaacTTATATTTAGTGGAT GCCAGagtcccattcgccacccgaaggtgttgcccagccggcgcc
sg6	sgRNA targeting *TDH3*p at -541 relative to the TSS	F:ggcgccggctgggcaacaccttcgggtggcgaatgggactTACATGCCCA AAATAGGGGGgttttagagctagaaatagcaagttaaaataaggctagtc<br>R:gactagccttattttaacttgctatttctagctctaaaacCCCCCTATTTTGGG CATGTAagtcccattcgccacccgaaggtgttgcccagccggcgcc
sg7	sgRNA targeting *HRK1*p at -41 relative to the TSS	F:ggcgccggctgggcaacaccttcgggtggcgaatgggactAAGTGGAAAA CTGTTAAATCgttttagagctagaaatagcaagttaaaataaggctagtc<br>R:gactagccttattttaacttgctatttctagctctaaaacGATTTAACAGTTTT CCACTTagtcccattcgccacccgaaggtgttgcccagccggcgcc
sg8	sgRNA targeting at -213 relative to the TSS	F:ggcgccggctgggcaacaccttcgggtggcgaatgggactTAAGCGGTTG GGAACTGTCTgttttagagctagaaatagcaagttaaaataaggctagtc<br>R:gactagccttattttaacttgctatttctagctctaaaacAGACAGTTCCCAAC CGCTTAagtcccattcgccacccgaaggtgttgcccagccggcgcc
sg9	sgRNA targeting at -355 relative to the TSS	F:ggcgccggctgggcaacaccttcgggtggcgaatgggactAAGGGGCGG CGACGACGTAGgttttagagctagaaatagcaagttaaaataaggctagtcR: gactagccttattttaacttgctatttctagctctaaaacCTACGTCGTCGCCGC CCCTTagtcccattcgccacccgaaggtgttgcccagccggcgcc

sg10	sgRNA targeting *SSK2*p at -20 relative to the TSS	F:ggcgccggctgggcaacaccttcgggtggcgaatgggactCCAAATCTGCAGATTAGAAGgttttagagctagaaatagcaagttaaaataaggctagtc R:gactagccttattttaacttgctatttctagctctaaaacCTTCTAATCTGCAGATTTGGagtcccattcgccacccgaaggtgttgcccagccggcgcc
sg11	sgRNA targeting *SSK2*p at -183 relative to the TSS	F:ggcgccggctgggcaacaccttcgggtggcgaatgggactCGTTTCATACCCTGAAACAGgttttagagctagaaatagcaagttaaaataaggctagtc R:gactagccttattttaacttgctatttctagctctaaaacCTGTTTCAGGGTATGAAACGagtcccattcgccacccgaaggtgttgcccagccggcgcc
sg12	sgRNA targeting *SSK2*p at -320 relative to the TSS	F:ggcgccggctgggcaacaccttcgggtggcgaatgggactTCGTCCAATTCCATCTCTACgttttagagctagaaatagcaagttaaaataaggctagtc R:gactagccttattttaacttgctatttctagctctaaaacGTAGAGATGGAATTGGACGAagtcccattcgccacccgaaggtgttgcccagccggcgcc
sg13	sgRNA targeting *BDH2*p at -32 relative to the TSS	F:ggcgccggctgggcaacaccttcgggtggcgaatgggactTCATTCTTGAACCTTGTCATgttttagagctagaaatagcaagttaaaataaggctagtc R:gactagccttattttaacttgctatttctagctctaaaacATGACAAGGTTCAAGAATGAagtcccattcgccacccgaaggtgttgcccagccggcgcc
sg14	sgRNA targeting *BDH2*p at -177 relative to the TSS	F:ggcgccggctgggcaacaccttcgggtggcgaatgggactAGCAAGGGGGTTAACCACCAgttttagagctagaaatagcaagttaaaataaggctagtc R:gactagccttattttaacttgctatttctagctctaaaacTGGTGGTTAACCCCCTTGCTagtcccattcgccacccgaaggtgttgcccagccggcgcc
sg15	sgRNA targeting *BDH2*p at -321 relative to the TSS	F:ggcgccggctgggcaacaccttcgggtggcgaatgggactAGAAACTGAAATCCCTTCCGgttttagagctagaaatagcaagttaaaataaggctagtc R:gactagccttattttaacttgctatttctagctctaaaacCGGAAGGGATTTCAGTTTCTagtcccattcgccacccgaaggtgttgcccagccggcgcc
sg16	sgRNA targeting *ISC1*p at +24 relative to the TSS	F:ggcgccggctgggcaacaccttcgggtggcgaatgggactGTTCACGAGAGGAAGGAAGAgttttagagctagaaatagcaagttaaaataaggctagtc R:gactagccttattttaacttgctatttctagctctaaaacTCTTCCTTCCTCTCGTGAACagtcccattcgccacccgaaggtgttgcccagccggcgcc
sg17	sgRNA targeting *ISC1*p at -143 relative to the TSS	F:ggcgccggctgggcaacaccttcgggtggcgaatgggactATGAGCGGTTTTAATTGAATgttttagagctagaaatagcaagttaaaataaggctagtc R:gactagccttattttaacttgctatttctagctctaaaacATTCAATTAAAACCGCTCATagtcccattcgccacccgaaggtgttgcccagccggcgcc
sg18	sgRNA targeting *ISC1*p at -233 relative to the TSS	F:ggcgccggctgggcaacaccttcgggtggcgaatgggactTTTGCTGACGTGCCAGTACCgttttagagctagaaatagcaagttaaaataaggctagtc R:gactagccttattttaacttgctatttctagctctaaaacGGTACTGGCACGTCAGCAAAagtcccattcgccacccgaaggtgttgcccagccggcgcc
sg_ PDR12	sgRNA targeting *PDR12* at +668 relative to the TSS	F: gactgaaatgatgtctaagtataa R:aaacttatacttagacatcatttc
sg_ HRK1	sgRNA targeting *HRK1* at +32 relative to the TSS	F: gactttgtcgagaaacccattcca R: aaactggaatgggtttctcgacaa
sg_ SSK2	sgRNA targeting *SSK2* at -221 relative to the transcription start site	F: tctaaggcaataacacactg R: cagtgtgttattgccttaga

Table S9. Primers used for verification of sgRNA integration and strain construction.

**Description**	**Primer sequences (5'->3')**
Primer set for verification of *in vivo* assembly of sg1 in level 2 plasmids	F: ggatgtatgggctaaatgtacggg<br>R: acacacataaacaaacaaaagttttagagct
Primer set for verification of *in vivo* assembly of sg2 in level 2 plasmids	F: ggatgtatgggctaaatgtacggg<br>R: aataagtatataaagacggtgttttagagctagaaat
Primer set for verification of *in vivo* assembly of sg3 in level 2 plasmids	F: ggatgtatgggctaaatgtacggg<br>R: actctggagtaaatgatgacacagtt
Primer set for verification of *in vivo* assembly of sg4 in level 2 plasmids	F: ggatgtatgggctaaatgtacggg<br>R: tagcgcaactacagagaacagttttagag
Primer set for verification of *in vivo* assembly of sg5 in level 2 plasmids	F: ggatgtatgggctaaatgtacggg<br>R: ctggcatccactaaatataagttttagagc
Primer set for verification of *in vivo* assembly of sg6 in level 2 plasmids	F: ggatgtatgggctaaatgtacggg<br>R: tacatgcccaaaataggggggtt
Primer set for verification of *in vivo* assembly of sg7 in level 2 plasmids	F: ggatgtatgggctaaatgtacggg<br>R: aagtggaaaactgttaaatcgttttagagc
Primer set for verification of *in vivo* assembly of sg8 in level 2 plasmids	F: ggatgtatgggctaaatgtacggg<br>R: taagcggttgggaactgtctg
Primer set for verification of *in vivo* assembly of sg9 in level 2 plasmids	F: ggatgtatgggctaaatgtacggg<br>R: aaggggcggcgacga
Primer set for verification of *mRuby2* integration at the *PDR12* locus	F: gatggtctggaccaaagcga<br>R: aataagcacctgccgtggtg
Primer set for verification of *HRK1* locus replacement by Venus	F: ccgtcaataacgtagcctttcttt<br>R1: aatggtcagctaattgaacaccacc<br>R2: cctctgtgctgttgactcga
Primer set for verification of *SSK2* locus disruption	F: tgttccaaattacctacaactcgct<br>R: aaactcttcctccgtgctcc
Primer set for verification of *dCas9* presence in the CRISPRa/i plasmid	F: ggagattagcggggtcgaag<br>R: aggggagagccagctcatta
Primer set for cPCR verification (*ADE2* amplification)	F: taaaaccgcacatgcggca<br>R: taggagggggacaattggg
